# Pathogenicity of *Mycobacterium tuberculosis* Is Expressed by Regulating Metabolic Thresholds of the Host Macrophage

**DOI:** 10.1371/journal.ppat.1004265

**Published:** 2014-07-24

**Authors:** Parul Mehrotra, Shilpa V. Jamwal, Najmuddin Saquib, Neeraj Sinha, Zaved Siddiqui, Venkatasamy Manivel, Samrat Chatterjee, Kanury V. S. Rao

**Affiliations:** Immunology Group, International Centre for Genetic Engineering and Biotechnology, New Delhi, India; Harvard School of Public Health, United States of America

## Abstract

The success of *Mycobacterium tuberculosis* as a pathogen derives from its facile adaptation to the intracellular milieu of human macrophages. To explore this process, we asked whether adaptation also required interference with the metabolic machinery of the host cell. Temporal profiling of the metabolic flux, in cells infected with differently virulent mycobacterial strains, confirmed that this was indeed the case. Subsequent analysis identified the core subset of host reactions that were targeted. It also elucidated that the goal of regulation was to integrate pathways facilitating macrophage survival, with those promoting mycobacterial sustenance. Intriguingly, this synthesis then provided an axis where both host- and pathogen-derived factors converged to define determinants of pathogenicity. Consequently, whereas the requirement for macrophage survival sensitized TB susceptibility to the glycemic status of the individual, mediation by pathogen ensured that the virulence properties of the infecting strain also contributed towards the resulting pathology.

## Author Summary

*Mycobacterium tuberculosis* (Mtb) is a highly successful human pathogen, representing the leading bacterial cause of death worldwide. Mtb infects macrophages and it adapts to the hostile intracellular milieu of this cell by exploiting the plasticity of its central carbon metabolism machinery. While several studies have detailed the bacterial adaptations that accompany infection, it is still unclear whether this process also involves engagement with host metabolic pathways. We therefore profiled the kinetic flux of host cell metabolites in macrophages that were infected with differently virulent Mtb strains. Interestingly, we found that Mtb pathogenicity was indeed intimately linked to its capacity to regulate host cell metabolism. A unique subset of host pathways was targeted so as to integrate the glycolytic threshold governing macrophage viability with mechanisms ensuring intracellular bacterial survival. Perturbation of macrophage glycolytic flux was enforced through pathogen-induced enhancement in glucose uptake, which in turn was also influenced by the extracellular glucose concentration. This observation rationalizes the increased susceptibility of diabetic individuals to TB infection Interestingly, Mtb strains also differed in their capacities to stimulate macrophage glucose uptake. Consequently, the resulting pathology is likely dictated both by the individual's glycemic status, and the nature of the infecting strain.

## Introduction

Pathogenicity of *Mycobacterium tuberculosis* (Mtb) has been attributed to the plasticity of its central carbon metabolism (CCM) machinery, which facilitates ready adaptation of pathogen to the intracellular milieu of the macrophage [1]. Emerging evidence, however, suggests that Mtb pathogenicity is also supported by engagement with metabolic pathways of the host cell. Thus while Mtb adaption to host requires the switch in bacterial CCM towards catabolism of host lipid substrates [2]–[5], optimal exploitation of this switch involves pathogen-induced promotion of lipid body (LB) accumulation by the host macrophage [6]–[8]. This ensures an abundant supply of the lipid substrates.

To investigate how Mtb infection influences CCM of the host macrophage we examined time-dependent modulations in macrophage metabolism, after infection with mycobacterial strains that varied in both genotype and phenotype. The resulting data, describing the temporal effects against a gradient of mycobacterial virulence, confirmed that Mtb pathogenicity was indeed intimately linked to its capacity to regulate host cell metabolism. Further, we also discovered that expression of virulence required the pathogen to engage with a unique subset of host metabolic pathways. Characterization of these pathways revealed that metabolic thresholds governing host cell survival were tightly assimilated with mechanisms regulating intracellular survival of the bacilli. This synthesis then provided the framework for convergence of both host- and pathogen-derived factors, in dictating the pathology of Mtb infection.

## Results

### Profiling the metabolic flux of macrophages

For these studies we primarily employed PMA-differentiated macrophage like THP-1 cells. To characterize the CCM of these cells, and also subsequently delineate the effects of Mtb infection, we adopted the procedure of kinetic flux profiling. In this procedure cells are fed with an isotopically labeled carbon source, followed by a determination of the rates at which this label is then incorporated into the downstream metabolites [9]–[11]. Kinetic profiling possesses the advantage of being sensitive to even subtle effects on host cell metabolism. Importantly for our purpose, it also circumvented the potential complication of host metabolites being contaminated with contributions from the pathogen either as a result of export or diffusion, or, simply leakage from bacilli during the extraction procedure. This could be confirmed in initial experiments where cells infected with the Mtb strain H37Rv were labeled with ^13^C_6_-glucose, and label incorporation into both host- and bacterial-derived metabolites was compared. We found that the labeling rates of host metabolites were between 40- to >100-fold higher than that of the corresponding bacterial counterparts (Figure S1). Thus, kinetic profiling enabled us to specifically monitor metabolite flux in the host cell, without interference from bacterial products or processes.

Before examining the effects of infection, it was necessary for us to first characterize the metabolic flux in uninfected THP-1 cells. For this we pulse-fed cells with ^13^C_6_-glucose for various times, and then employed liquid chromatography-tandem mass spectrometry (LC-MS/MS) to measure rates of label incorporation in a total of thirty-five metabolites derived either from the glycolytic, the pentose phosphate (PP), or nucleotide flux pathways. In addition, the tricarboxylic acid (TCA) cycle and the citrate shuttle pathway leading to lipid biosynthesis were also followed. Rapid incorporation of ^13^C-label was detected in glucose-6-phosphate (G6P), the first intermediate in glycolysis, with its subsequent utilization then being distributed between the glycolytic and PP pathways. The latter however primarily fed back into glycolysis at either fructose-1,6-bisphosphate (FBP) or glyceraldehyde-3-phosphate (G3P) since, apart from ribose-5-phosphate (R5P), no labeling of downstream intermediates in either nucleotide or tryptophan synthesis was detected.

**Figure 1:**
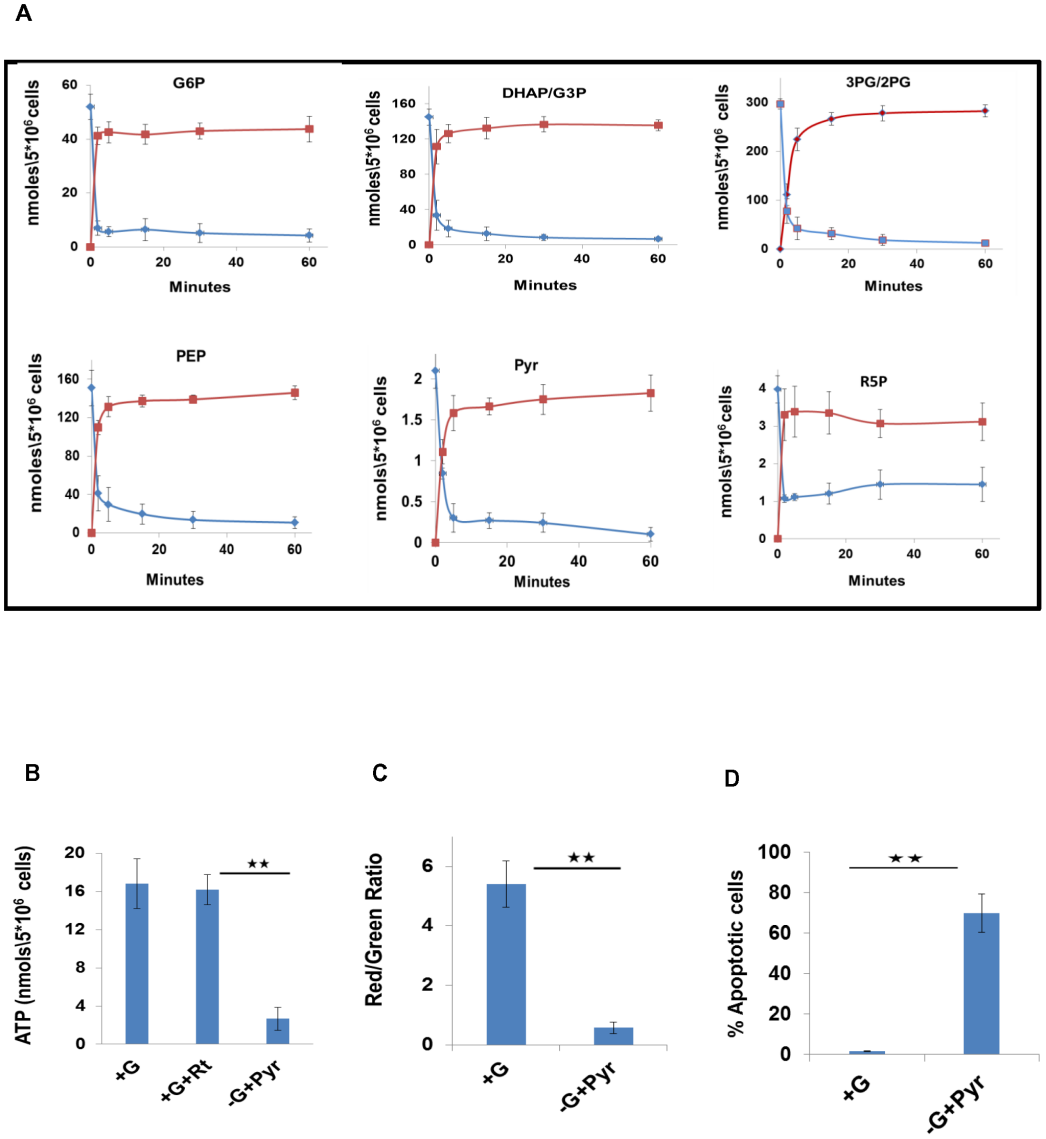
Metabolic profiling of PMA-differentiated THP-1 cells and their dependence on glycolysis. A. Determining biosynthetic rates of THP-1 cell metabolites. Cells were labeled with ^13^C_6_-glucose, and label incorporation in metabolites of the CCM pathways was monitored at 0, 2, 5, 15, 30 and 60 minutes post label addition. Profiles for both ^13^C-incorporation (red line) and consumption of the naturally occurring isotopomer (blue line) for a representative set of intermediates are shown here (for values, n = 3, mean ±SD). B. Uninfected cells were maintained in complete RPMI media either in the absence (+G), or presence (+G+Rt) of rotenone. A parallel set of cells was maintained in glucose free media supplemented with 1 mM pyruvate as a feeder for the TCA cycle (-G+Pyr). Intracellular ATP levels were then determined by LC-MS/MS (n = 3, mean ±SD; * *p≤0.01) C. Maintenance of the mitochondrial membrane potential is regulated by the glycolytic flux. Uninfected cells were maintained either in complete RPMI media (+G) or in Glucose free media supplemented with Pyruvate as a feeder for the TCA cycle (-G+Pyr). At 24 hr after treatment, cells were stained with JC-1 and the mitochondrial membrane potential was determined in terms of the red/green fluorescence ratio by confocal microscopy (n = 3, mean ±SD, **p≤0.01). D. The proportion of cells undergoing apoptosis was also determined and the results are presented (n = 3, mean ±SD, **p≤0.01).

Labeling of the glycolytic intermediates proceeded rapidly all the way up to pyruvic acid (Pyr), which was then converted into acetyl-CoA (AcCoA) for subsequent condensation with oxaloacetate (OA) to generate the tricarboxylic acid cycle (TCA) intermediate ^13^C_2_-citric acid. Consistent with earlier reports for macrophages though [12] , we found activity of the TCA cycle to be markedly dampened in these cells. No detectable labeling was observed in intermediates downstream of citrate, all the way up to OA. Further, contributions from anaplerotic reactions or gluconeogenesis was also not detected. In line with our previous finding [13], there was no significant conversion of AcCoA into the ketone body 3-hydroxybutyric acid (3HB). Further, no evidence for any *de novo* synthesis of either free fatty acids (FA) or cholesterol (CL) was also found. ^13^C-Labeling profiles of representative metabolites are shown in Figure 1A, while the results are summarized in Figure S2A&S2B.

### Glycolytic dependence of THP-1 cells

Our observation that the TCA cycle activity was attenuated in these cells was intriguing as it suggested that ATP synthesis by the oxidative phosphorylation pathway may also be diminished. This latter aspect could be experimentally confirmed by demonstrating that addition of the electron transport chain inhibitor Rotenone to cells had no significant effect on the intracellular ATP level (Figure 1B). Further, separation of the mitochondrial and cytoplasmic fraction of the cells revealed that over 85% of the total cellular ATP was present in the cytoplasm (Figure S3A). This finding is consistent with the fact that the ATP was derived, at least primarily, from the glycolytic rather than the oxidative phosphorylation pathway.

A significant consequence of the attenuated TCA cycle, and the resultant oxidative phosphorylation, activity was that maintenance of cell viability was now largely dependent upon the ATP generated through glycolysis. This was evident from experiments where depletion of glucose from the culture medium - after supplementing the latter with Pyr as the mitochondrial energy source - caused ∼70% reduction in cellular ATP levels (Figure.1B). Importantly, this then also led to a loss in mitochondrial membrane potential (ΔΨ_m_) (Figure. 1C), and the consequent activation of apoptotic death (Figure. 1D). Similar effects were produced, in Pyr-supplemented media, by the glycolysis inhibitors 5-thioglucose (5TG) and 2-deoxyglucose (2DG), and the GAPDH inhibitor 3-bromopyruvate (3BP) [14], [15]. The latter finding is notable since GAPDH inhibition mitigates the payoff phase of glycolysis that is responsible for generating the net gain in both ATP and NADH (Figure S3B–S3D). These collective results, therefore, establish that THP-1 cells are indeed glycolytic in nature, and that their preservation in a viable state requires maintenance of an active flux through the payoff phase of glycolysis.

### A coupled Ordinary Differential Equation (ODE) model for the macrophage CCM

To capture the overall metabolic flux distribution in these cells we mathematically modeled the CCM by using a system of coupled ODEs that encompassed the relationship between individual metabolites in the pathway [16]. In this model we incorporated the experimentally determined rates of actual synthesis and consumption of each metabolite, to estimate the parameters that yielded metabolite steady state concentrations that corresponded with the experimentally determined values. For determination of rates of individual reactions we took the results from the experiment described in Figure 1A and first calculated the ^13^C-label incorporation rates for each metabolite. This was measured as the slope of the plot obtained up to the half-maximal value of ^13^C-incorporation in each case. Similarly, the slope for disappearance (i.e. consumption) of the corresponding naturally occurring isotopomer was also determined for each metabolite. The ^13^C-label incorporation rate of a given metabolite (e.g. X) gives its net rate of synthesis, which represents the combined outcome of both synthesis and consumption of that molecule (i.e. ^13^C-labeling rate of X =  actual rate of synthesis – rate of consumption). Therefore, the actual rate of synthesis of ‘X’ (r_X_) could then be calculated as: rate of ^13^C-incorporation in X+ rate of decrease in concentration of its ^12^C-labeled counterpart. Taking the actual rates of synthesis and consumption then, we could also estimate the steady state concentration of each metabolite. The method employed for this is described in Figure S3E.

**Figure 2:**
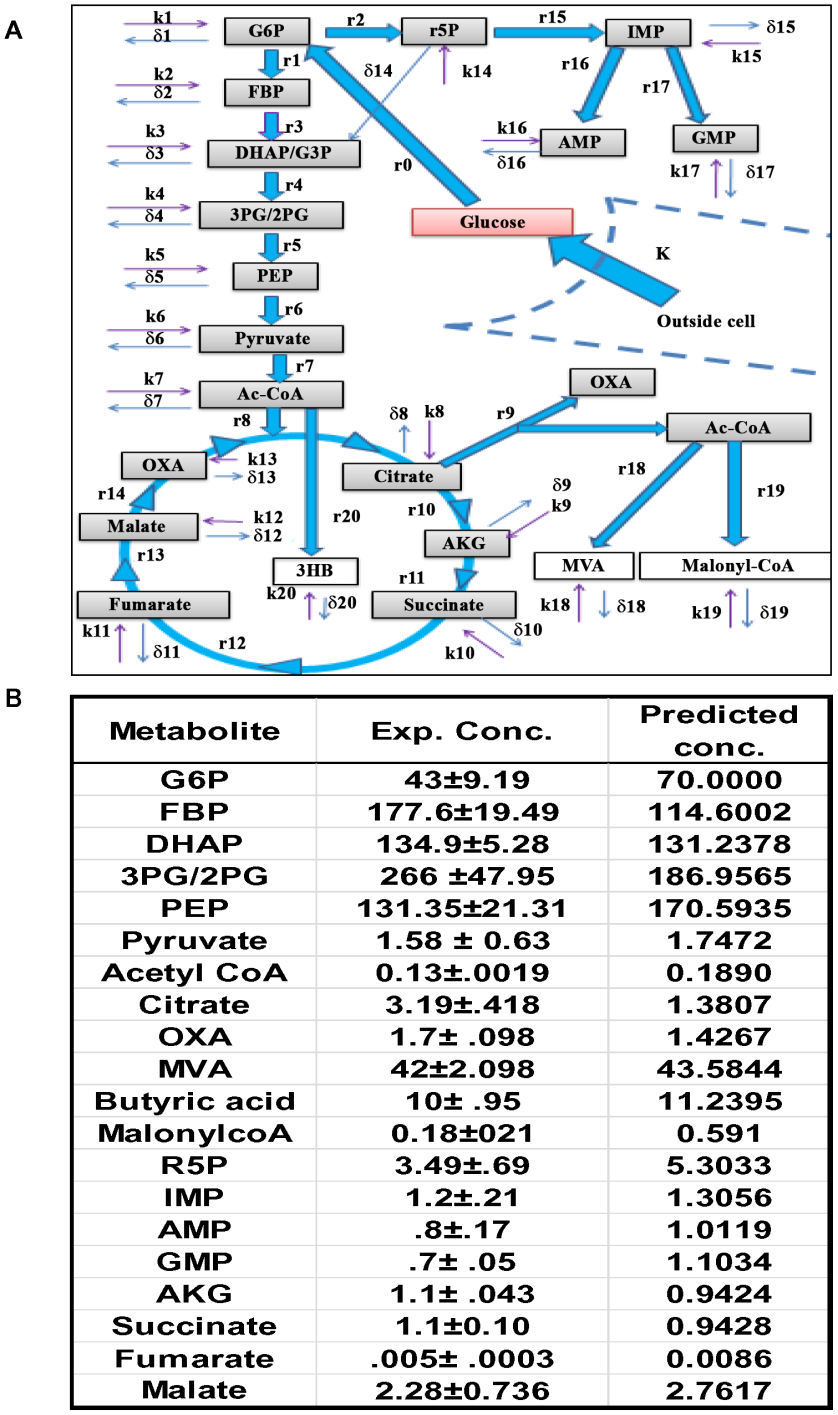
The CCM pathways captured by the ODE model. A. Figure depicts the uptake of glucose by the cells (K, rate of uptake), and its subsequent recruitment into the CCM pathways. The latter is initiated by the conversion of glucose into G6P, which then feeds both the PP and the glycolysis pathways. The integration of glycolysis with the TCA cycle is also shown, as well as the generation of 3HB from mitochondrial AcCoA. The cataplerosis of mitochondrial citrate and its subsequent breakdown to generate the cytoplasmic pools of OXA (OA) and AcCoA is also depicted. Cytoplasmic AcCoA can then potentially initiate CL biosynthesis through the intermediate MVA, and/or FA synthesis through the Malonyl-CoA (MaCoA) pathway. In this scheme r(n) represents the actual rate of synthesis of the corresponding metabolic intermediate and was calculated as described in the text. The term k(n) denotes unknown fluxes that may contribute additional input to the metabolite concentration (i.e. inflow). Side-reactions that could provide for outflow of a given metabolite from the CCM pathways are accounted for by the term δ(n). The detailed set of equations employed in the model is provided in Methods S1. B. The steady state concentrations of metabolites predicted by our model are compared with the experimentally (Exp. Conc.) determined values (n = 5, ±S.D.) as described in the text.

Figure 2A shows the pathway that was modeled and the flux distribution parameters that were estimated. The resulting model consisted of twenty-one metabolic state variables and sixty-one rate parameters. Of the latter, thirty-eight were those that were experimentally determined thus ensuring the robustness of the model. Fidelity of the model parameters was established by rigorous validation (Methods S1, Table S6–S9), which was also further supported by the fact that the model-derived metabolite steady state concentrations matched well with the experimentally determined values (Figure. 2B).

### Infection-induced effects on host cellular glucose uptake

To study the effects of infection on macrophage CCM, we took a panel of five mycobacterial strains. In addition to *Mycobacterium smegmatis* (M.smeg), it included the Mtb strains H37Ra, H37Rv, BND433, and JAL2287. While H37Ra and H37Rv are the respective avirulent and virulent counterparts of a laboratory strain, BND433 and JAL2287 are clinical isolates of the CAS lineage [13], [17]. Following infection in THP-1 cells, M.smeg was rapidly cleared by 24 hr post-infection (p.i.), whereas the remaining strains persisted at a bacillary load that was of the order: JAL2287>BND433>H37Rv>>H37Ra (Figure S4A). Further, while H37Rv-, JAL2287-, or BND433-infected cells displayed significant - but variable - extents of necrotic death by 72 hr p-i, H37Ra-infected cells primarily underwent apoptosis whereas cells infected with M.smeg remained viable after bacterial clearance (Figure S4B). Thus, given that the mycobacterial panel encompassed distinct phenotypic properties that ranged from non-pathogenic to attenuated to differing degrees of virulence, a comparative analysis of the strain-specific effects was expected to yield insights into regulatory features that mediated mycobacterial virulence.

**Figure 3:**
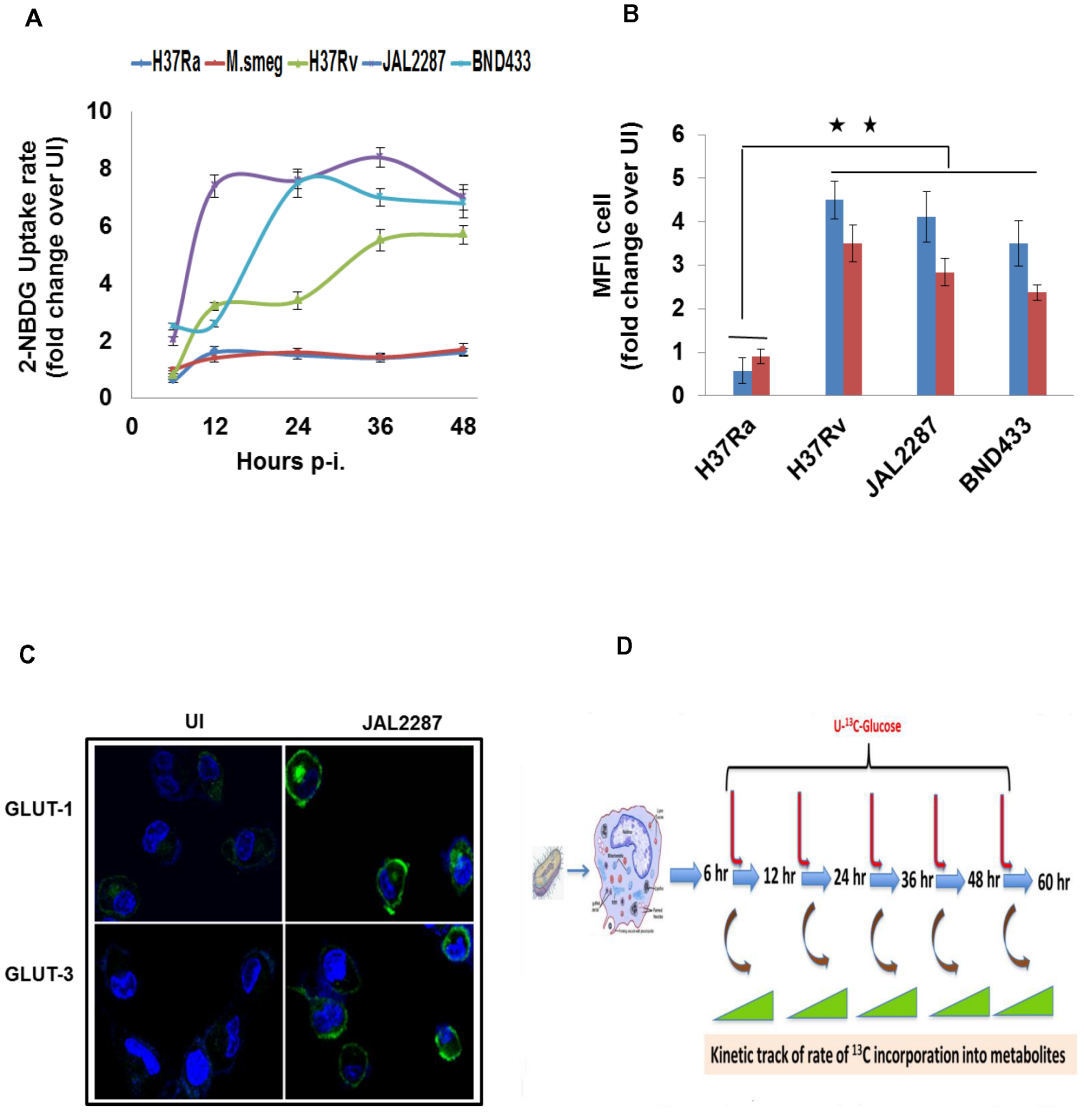
Potentiation of host cellular glucose uptake by Mtb infection. A. Infection-induced alterations in the glucose uptake capacity of THP-1 cells. Shown are the rates of uptake of 2-NBDG by infected cells, as a ratio of the corresponding value in uninfected (UI) cells, when measured at the indicated p-i time points. Values are the mean ±SD of three experiments. B. Potentiation of glucose uptake results from enhanced surface expression of glucose transporters GLUT-1 (blue bars) and GLUT-3 (red bars). Expression of the corresponding proteins on the surface of infected cells was detected by confocal microscopy after staining with the specific antibodies. Data was collected for between 60–80 cells per experiment in two separate experiments, and values are presented as fold change in MFI/cell over that obtained in uninfected (UI) cells. Results for a representative experiment (mean ±S.E.) are shown here. **p<0.01. While the results in M.smeg-infected cells are not shown, there was no detectable increase in levels of either of these proteins compared to that in UI cells (see also Figure S4C and S4D). C. Panel shows the confocal microscopy images comparing expression of GLUT-1 and GLUT-3 (green) in UI versus JAL2287 infected cells as a representative example. Cell nuclei were stained with DAPI (blue). D. Illustration of the experimental design for monitoring temporal modulations in metabolic flux of infected cells. Cells were infected with the individual strains of mycobacteria and, at the indicated times p-i, they were pulsed with ^13^C_6_-Glucose. Rates of the individual metabolic reactions were then determined by measuring the extent of label incorporation achieved at 2, 5, 15, 30, and 60 min after initiation of the pulse, at each of the infection times indicated. ^13^C-label incorporation in the individual metabolites was measured by LC-MS/MS tandem mass spectrometry.

We first monitored for any influence of infection on host cellular glucose uptake. For this THP-1 cells were infected with the individual mycobacterial strains, and the rate of uptake of 2-NBDG - a fluorescent analog of glucose – was measured at appropriate time intervals that spanned a total period of 48 hrs. Infection with M.smeg or H37Ra caused only a marginal effect on 2-NBDG uptake rates (Figure. 3A). In contrast, however, cells infected with either of the virulent Mtb (virMtb) strains exhibited a marked increase in the rate of glucose uptake. Depending upon the infecting strain and time p-i, this increase ranged from 2- to 8-fold greater than the rate seen in uninfected cells (Figure 3A). Subsequent experiments revealed that increased glucose uptake by virMtb infected cells was due to pathogen-induced enhancement in cell surface expression of the high affinity glucose transporters Glut1 and Glut3. This effect was seen in both THP-1 (Figure 3B&C) and primary human monocyte-derived macrophages (HuMΦ) (Figure S4C and S4D), but only after virMtb infection. No such response was induced by either H37Ra or M.smeg infection (Figure 3B), thereby explaining the specificity of the effect in Figure 3A.

### Mycobacterial infection perturbs host cell glycolysis

**Figure 4:**
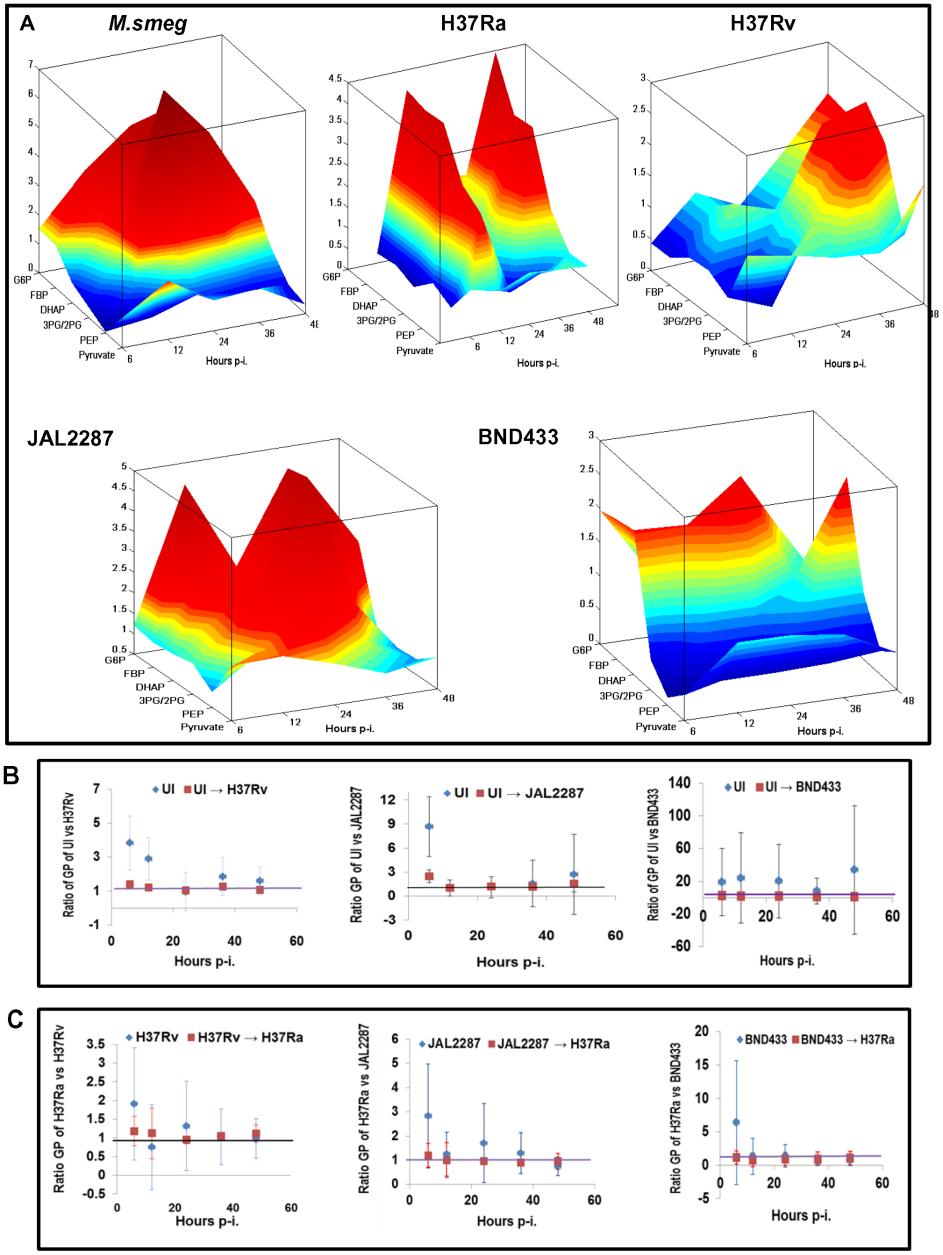
Delineating the virMtb-specific effects on macrophage glycolysis. A. Landscape representation of the temporal modulations in glycolytic flux in infected cells, relative to that in UI cells. The z-axis indicates the metabolites monitored in the glycolytic pathway, whereas the x-axis gives the time point of measurement p-i. The y-axis gives the actual rate of synthesis of each intermediate, as a ratio of that obtained in uninfected cells, at each of the infection times monitored. B. Panel depicts the ratio between the glycolytic profile (GP) of UI cells and that of the different virMtb-infected cells at the indicated time p-i. For this the ratio of the synthesis rates of each metabolite in UI versus the infected cells was determined. The mean and S.D. values of these ratios was then plotted to obtain distribution of the GP over time (see Methods S1). Points in blue refer to unperturbed UI cells whereas those in red reflect the profile of UI cells obtained upon changing the rate of glucose uptake and DHAP/G3P synthesis (details in Methods S1). C. Shown here is the simulated conversion of the GP of virMtb-infected cells into that seen in cells infected with H37Ra. The methodology followed was similar to that described for (B).

To determine the downstream consequences of the effects on glucose uptake, we took infected cells and pulse-fed them with ^13^C_6_-glucose at the times indicated in Figure 1A. Following this, we profiled the kinetic flux of the ^13^C label across the individual metabolites. The experimental design is illustrated in Figure 3D, and this strategy enabled us to determine the rates of individual reactions - at the various times p-i - in cells infected with each of the mycobacterial strains (Table S10). This experiment produced a complex data set that, unfortunately, was difficult to interpret. This was because each strain produced a unique glycolytic landscape profile with no distinct pattern segregating the effects of virulent Mtb strain from those of the non-pathogenic ones (Figure 4A).

Therefore, to interrogate these results further, we employed our ODE model for the infected cells across all the time points studied (see Figure 2A). Our aim here was to search for any underlying chokepoint reactions that may distinguish perturbations caused by the virMtb strains. That is we asked whether the glycolytic perturbations seen in Figure 4A were truly incoherent in nature, or whether they instead derived from targeted but variable effects on a small subset of the overall reactions? To address this we performed two related sets of simulation exercises. In the first we took the parameter set obtained for uninfected cells, and determined the minimum number of changes that would be required to simulate the profile of metabolite concentrations generated by each of the virulent Mtb strains. Interestingly, we found that this could be achieved through calibrated enhancements in rates of only two of the steps in the glycolytic pathway. These steps were: (i) uptake of glucose by the host cell and (ii) generation of DHAP and G3P through cleavage of FBP (Figure 4B). Especially significant also was the converse finding that the glycolytic landscape profiles (GP) produced against the virulent Mtb infections could be reset to resemble the H37Ra-specific response simply by appropriately attenuating the rates of these same two steps (Figure 4C). Although the robustness of our model gave confidence in these inferences we, nevertheless, additionally tested for fidelity of the predictions. For this we simulated a progressive reduction in glucose uptake rate in JAL2287-infected cells, and determined the consequent effects on GP, and on synthesis of R5P, AcCoA and FA. We found that the resulting trends compared well with experimentally determined values in cells cultured in medium containing decreasing concentrations of glucose (Figure S5A).

Thus in contrast to at least H37Ra, virMtb isolates were distinguished by their ability to promote glucose uptake by the host macrophage, while at the same time also accelerating the last reaction in the preparatory phase of glycolysis. The magnitude and dynamics of these influences however differed across the dimensions of strain variability and time, and it was these differences that then accounted for the heterogeneity of the profiles in Figure 4A. It is pertinent to note here that the virMtb-induced acceleration of synthesis of DHAP/G3P is especially relevant given that these molecules initiate the downstream steps of the energy generating, or payoff, phase of glycolysis.

### Augmentation of aerobic glycolysis prevents activation of the host cellular apoptotic response to infection

**Figure 5:**
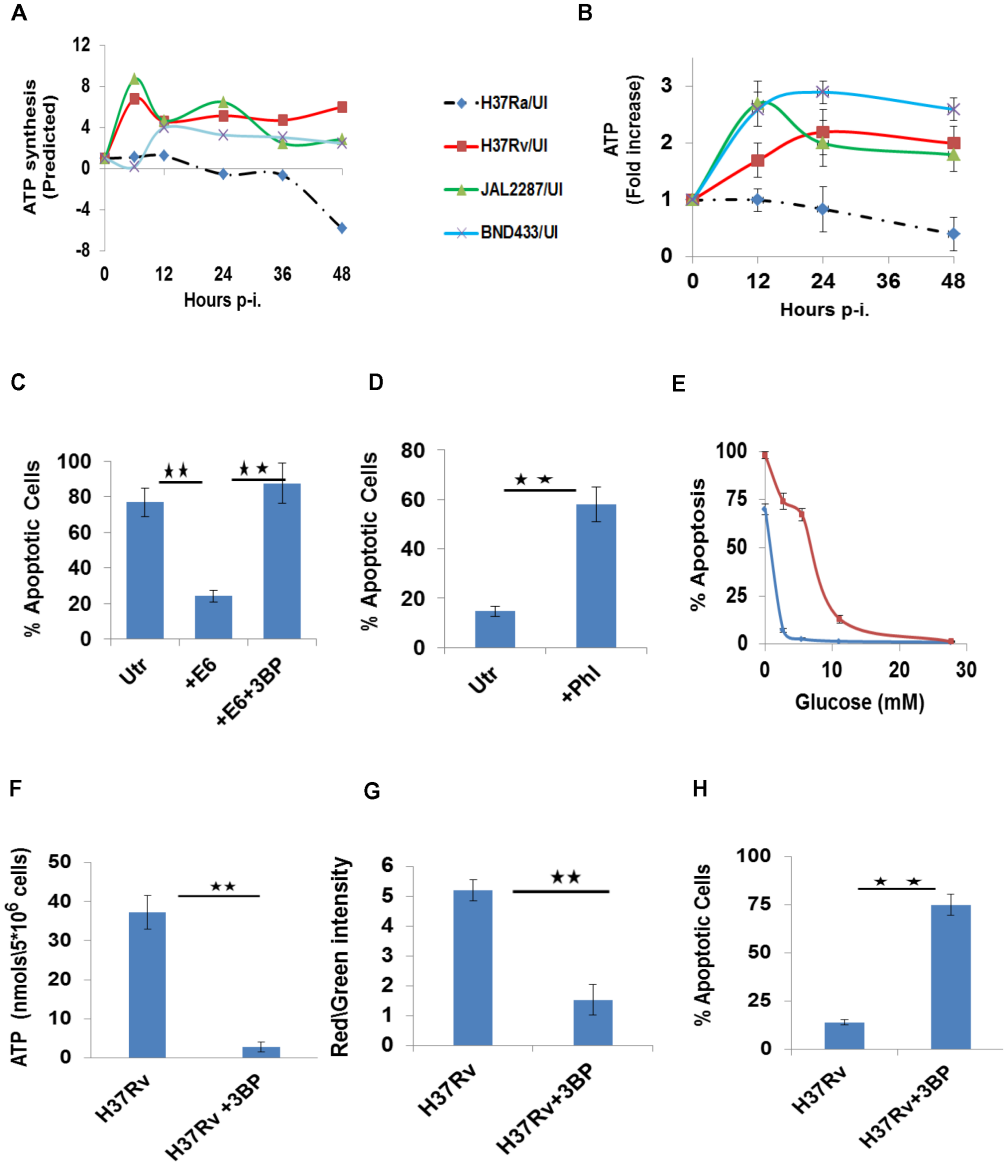
Perturbations in glycolytic flux control host cell death pathways. A. Estimated modulations in the cellular ATP synthesis rates, as a function of the duration of infection with the individual strains, are depicted as a ratio over the basal values in UI cells. Synthesis rates were calculated from the rates of the individual glycolytic reactions as described in the text. B. ATP levels were measured at the indicated times in infected cells by LC-MS/MS. Results obtained are profiled as a fold change in ATP concentrations over the corresponding level in UI cells, at each of the time points indicated. Values are the mean (±SD) of four experiments. C. H37Ra-infected THP-1 cells were either left untreated (Utr), or treated with 0.5 µM of the recombinant ESAT-6 protein (+E6) as described earlier [13]. A parallel set was also treated with the combination of ESAT-6 and 3BP (+E6+3BP). The proportion of apoptotic cells was determined at 72 hr p-i. in all cases (n = 3, ±S.D., significance, **p≤0.01). D. H37Rv- infected cells, were either left untreated (Utr), or treated with Phloretin (+Phl) at 20 µM. 72 hr later, the proportion of apoptotic cells was determined in all cases and the results (n = 3, ±S.D., significance, **p≤0.01) are presented here. E. Glucose-dependency thresholds of UI and H37Rv-infected macrophages. Either UI (blue line) or H37Rv-infected cells (red line) were cultured in medium containing the indicated amounts of glucose. Profiles give the percent of apoptotic cells, as a function of glucose concentration, when determined 48 hr later. Values are the mean (±S.D.) of three separate experiments. F-H. H37Rv-infected cells depend on glycolytic flux for ATP generation (F), and a maintenance of both mitochondrial membrane potential (G) and cell viability (H). H37Rv infected cells were left either untreated, or were treated with 3BP in medium supplemented with Pyr. The parameters described in the individual panels were measured at 48 hr and values in each panel are the mean (±S.D.) of between 3 to 5 experiments. (Significance, **p<0.01).

Consistent with expectations, the targeted perturbations exercised by virMtb strains markedly enhanced glycolytic synthesis of ATP by the host cell across all the time points studied. This was determined by calculating the net rate of ATP production in each instance, from the rates of the individual reactions in the preparatory and pay-off phases of glycolysis (Figure 5A). In the case of H37Ra infection however, the rate of host cellular ATP production progressively declined with a significant inhibition being evident by 48 hr (Figure 5A). These estimates of the net biosynthesis rates corresponded well with subsequent measurements of the intracellular ATP concentration (Figure 5B), thereby confirming that infection-induced perturbations in glycolysis indeed impacted on the overall energetics of the host cell.

Importantly, in keeping with the dependence of macrophage survival on glycolytically generated ATP, it was the depletion of the latter that caused apoptosis in H37Ra-infected cells. This aspect was verified by experiments where glucose uptake by these cells was potentiated by the exogenous addition of ESAT-6 as described earlier [13]. Thus, whereas ESAT-6 addition rescued H37Ra-infected cells from death, co-addition with 3BP to selective block glycolysis-dependent ATP generation – however – reversed this effect (Figure 5C).

In contrast to the situation with H37Ra, it was the endogenous augmentation of ATP biosynthesis that suppressed apoptosis of virMtb-infected macrophages. This was confirmed by our finding that either suppression of glucose transport with Phloretin [18]–[20], or depletion of glucose in the medium, induced apoptotic death of H37Rv-infected cells (Figure 5D&E). Also in line with the increased bioenergetic demand placed on infected macrophages, we found that maintenance of H37Rv-infected cells in a viable state required a higher threshold of glucose availability in comparison with that for uninfected cells (Figure 5E and Figure S5B). Finally, addition of 3BP to H37Rv-infected cells caused the expected suppression of cellular ATP synthesis (Figure 5F), resulting in mitochondrial membrane depolarization (Figure 5G) and apoptotic death (Figure 5H).

These collective results therefore establish the functional significance of pathogen-induced perturbations in glycolytic flux of the host macrophage. Importantly, by delineating the manner in which this influence impinged on the regulation of host cell apoptosis, these findings also unveil an early checkpoint that likely contributes towards pathogenesis of the infection.

### Virulence-specific perturbation of mitochondrial pathways

**Figure 6:**
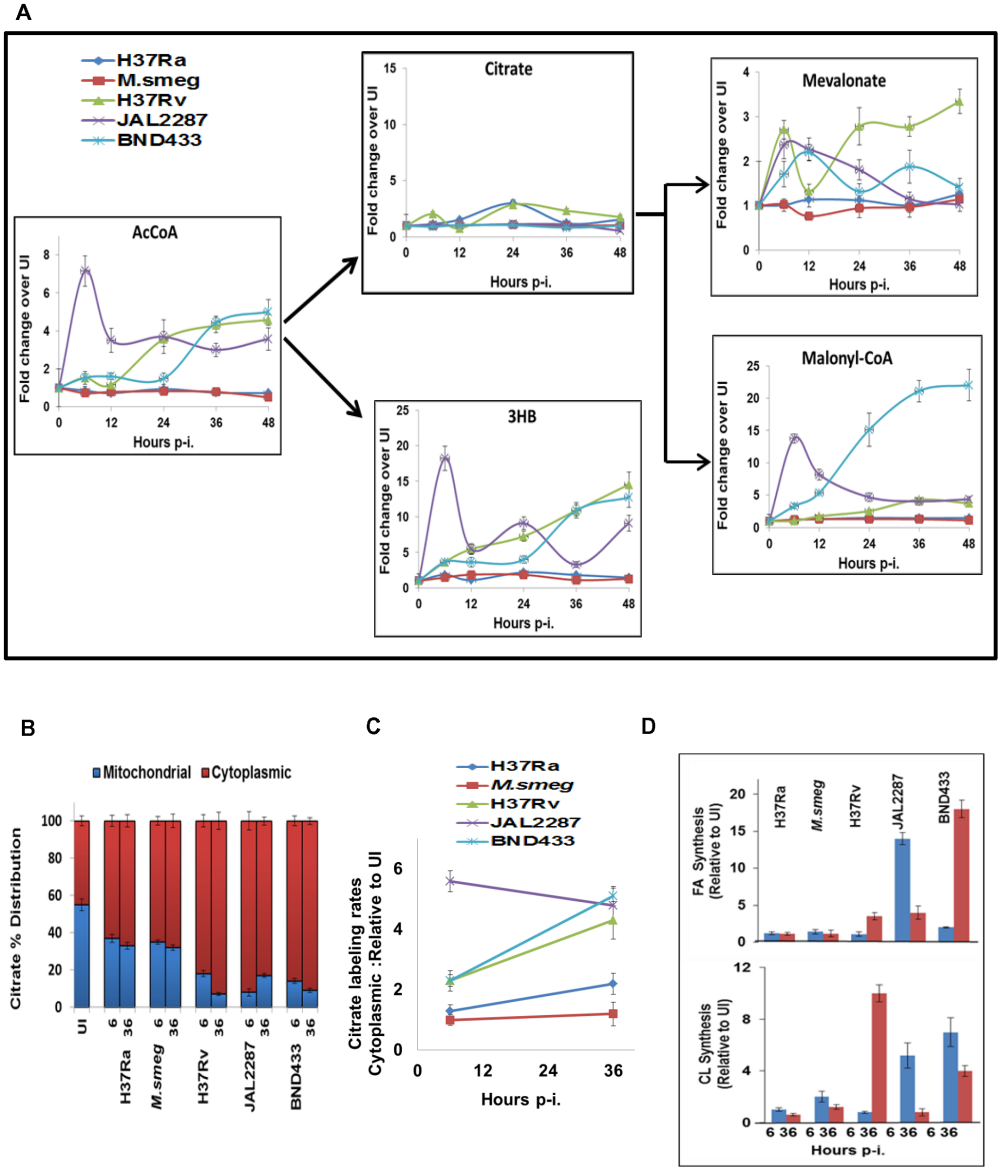
Citrate cataplerosis mediates *de novo* lipid synthesis in virMtb infected macrophages. A. Panel depicts the time-dependent effects of infection with the individual strains on AcCoA synthesis, followed by the downstream distribution of its utilization between citrate and 3HB synthesis. Also shown here, are the effects on citrate catabolism to produce the substrates for CL (Mevalonate) and FA (Malonyl-CoA) synthesis. Details are provided in the text and values are presented as fold increase in actual synthesis rates, over the corresponding rates in uninfected cells. These are the mean ±S.D of three independent determinations. B. Distribution of citrate between the cytoplasm and mitochondria at 6 and 36 hr p-i. Citrate concentration was determined by LC-MS/MS (n = 3, mean ±SD). C. Determining the rate of citrate export from mitochondria to cytoplasm. The rate of ^13^C-labeling of cytoplasmic citrate, at 6 and 36 hr p-i, was determined for each of the strains by quantifying rate of change in labeled cytoplasmic citrate pool after pulsing the infected cells with ^13^C labeled Glucose. The relative values, with respect to that in UI cells, are presented for each of the strains at 6 and 36 hours (n = 3, mean ±SD). D. virMtb infection stimulates *de novo* synthesis of FA and CL. Results at 6, and 36 hr p-i. are presented as the fold change in the percentage of ^13^C-label incorporation in newly synthesized palmitic acid and CL over that obtained in UI cells. The basal rate of ^13^C incorporation into FA and CL in UI cells was minimal (<10 pmole/min/3×10^7^ cells for palmitic acid and <5 fmol/min/3×10^7^ cells for CL).

Integration of glycolysis with the TCA cycle is mediated through oxidation of Pyr to AcCoA. Enzymatic condensation of this AcCoA with OA then generates the TCA cycle intermediate, citrate. Interestingly in this context, we found that virMtb-infected cells – but not H37Ra or M.smeg infected ones – also exhibited a pronounced increase in the rate of cellular AcCoA synthesis (Figure 6A). Strain-dependent differences were, however, noted in the kinetics of this process. JAL2287 infection produced an early burst of ∼7-fold increase, which then later subsided to about 3-fold over the basal value. In comparison, AcCoA production gradually increased in H37Rv-infected cells whereas BND433 infection yielded a biphasic response curve (Figure 6A). The mechanism involved, however, is presently unknown and awaits clarification.

Surprisingly, we found that the virMtb-induced augmentation of host cellular AcCoA synthesis did not translate into the expected increase in citrate production (Figure 6A). Instead, AcCoA was primarily utilized for synthesis of the ketone body 3HB (Figure 6A). Ketone body synthesis represents an alternate pathway for mitochondrial AcCoA, which is activated when AcCoA production rates exceed that of its utilization in the TCA cycle [21]. Thus, the observed conversion of accelerated AcCoA synthesis into 3HB generation probably reflects a consequence of the attenuated nature of the TCA cycle in these cells. Indeed we have previously demonstrated that activation of this host pathway constituted a virulence mechanism of Mtb, with 3HB then driving acquisition of the ‘foamy’ phenotype by the host macrophage [13].

### virMtb induces host cellular *de novo* lipid biosynthesis by activating citrate cataplerosis

Although virMtb infection did not affect citrate production we, nonetheless, observed significant differences at the level of MVA and MalonylCoA (MaCoA). Synthesis of both of these intermediates was heightened in virMtb- but not in H37Ra- or M.smeg-infected cells (Figure 6A). MVA and MaCoA, intermediates in the biosynthesis of CL and FA respectively, are both products of a cataplerotic pathway of the TCA cycle wherein mitochondrial citrate is exported to the cytoplasm. Enzymatic cleavage of this exported citrate by ATP-citrate lyase generates cytoplasmic AcCoA, which then constitutes the substrate for both MVA and MaCoA biosynthesis. To resolve how synthesis of these latter molecules was enhanced in the absence of any effect on citrate production, we pulse-labeled infected cells at 6 and 36 hr p-i. The cytoplasmic and mitochondrial fractions were then separated to determine the relative citrate concentrations present in each fraction. In addition we also monitored the rate of labeling of the cytoplasmic citrate pool, as a measure of the rate of its export from the mitochondria.

Figure 6B shows that the proportion of the mitochondrial citrate was significantly reduced in virMtb-infected cells. This effect was more pronounced at 36 hr, relative to 6 hr, for cells harboring either H37Rv or BND433 whereas the converse was true of those infected with JAL2287 (Figure 6B). These effects could subsequently be rationalized through a comparison of the relative rates of citrate export to the cytoplasm (Figure 6C). Exposure to virMtb uniformly led to an increase in this process, with an initial effect at 6 hr being further amplified by 36 hr in the case of H37Rv and BND433 infection. For JAL2287 however, peak activation of host cellular citrate export was already evident by 6 hr, with a slight decrease at 36 hr (Figure 6C). In contrast to the effects of virMtb, infection with M.smeg failed to exert any influence whereas only a marginal effect was detected in response to H37Ra infection (Figure 6C).

The functional significance of virMtb-specific augmentation in MVA and MaCoA synthesis could next be confirmed by experiments demonstrating that this process also resulted in a corresponding increase in synthesis rates of CL, and palmitic acid as a representative FA (Figure 6D). Interestingly, although for reasons presently unknown, infection either with JAL2287 or BND433 preferentially activated FA synthesis whereas H37Rv infection biased towards CL production (Figure 6D). Furthermore, the kinetics of FA induction also differed between JAL2287 and BND433 infected cells (Figure 6D). Nonetheless, given that intracellular survival and persistence of Mtb depends upon its ability to access host-derived FA and CL as nutrients [4], [22], [23], these results would suggest that activation of host lipid synthesis likely constitutes an obligatory prerequisite for establishment of infection.

### virMtb-mediated induction of ‘foamy’ macrophage (FM) differentiation

**Figure 7:**
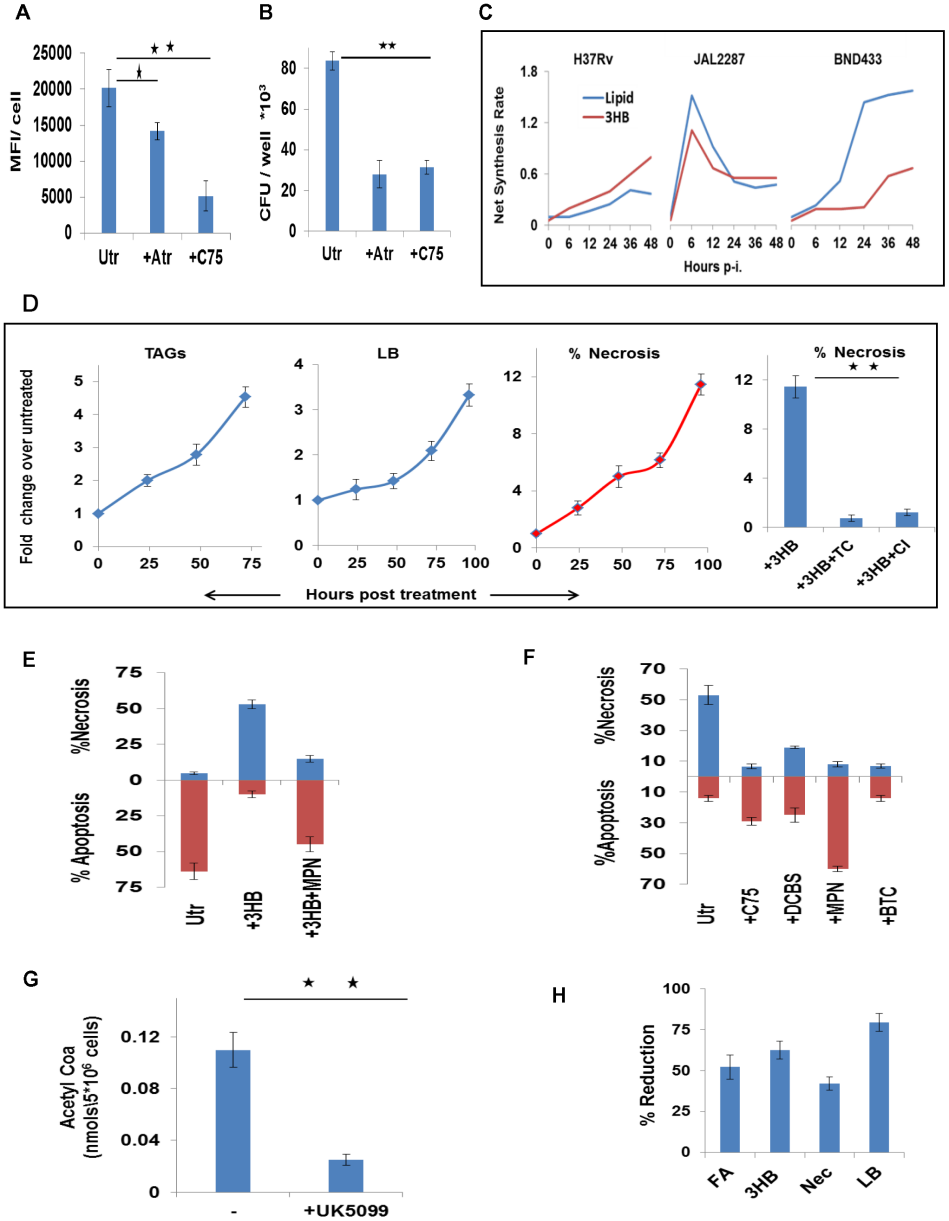
Foamy macrophage differentiation and the induction of necrosis. A. H37Rv-infected cells were treated either with C75 (20 µM), or Atorvastatin (Atr, 10 µM) and then scored for lipid bodies at 48 hr p-i. For this cells were stained with Lipid Tox and the mean fluorescence intensity (MFI) per cell was determined. Cell nuclei were counterstained with DAPI and data represents the MFI/cell where values are the mean ±SE obtained from at least 70 cells. Results from one of two separate experiments are shown (significance, *p<0.05; **p<0.01). Utr, untreated cells. B. H37Rv-infected cells were treated as described in (A). At 72 hr later, cells were lysed and plated for determination of the bacterial CFU values (n = 3 mean ±SE, significance **p<0.01). C. The time course profile of production of 3HB and lipids (i.e. FA+CL), as estimated by the ODE model, is shown for the virMtb infected cells. D. UI cells were either left untreated, or were treated with 3HB (100 µM). At the times indicated, these cells were scored for the accumulation of TAGs, and LBs, and induction of necrosis. The extreme right panel shows that 3HB-induced necrosis could be reversed by treatment of cells with either Triacin C (TC, 5 µM), or CI-976 (CI, 1 µM) an inhibitor of the LB biogenesis regulator Acyl-CoA cholesterol acyltransferase. Significance, **p<0.01. E. Comparison of the extent of necrosis in H37Ra-infected cells that were left either untreated (Utr) cells, treated with 3HB (100 µM) alone, or treated with a combination of 3HB and MPN (100 nM). The time point of measurement here was 48 hr p-i. (n = 3, mean ±SD). F. H37Rv infected cells were either left untreated (Utr) or treated either with C75 (20 µM), or the citrate transport inhibitor 1,2,3-benzenetricarboxylic acid (BTC) at 200 µM [57], or the citrate lyase inhibitor 3,5-dichloro-2-hydroxy-N-(4-methoxybiphen-3-yl) benzene sulfonamide (DCBS) at 50 µM [58], or MPN at 100 nM[13]. The extent of cell death either by apoptosis or necrosis was then scored at 48 hr p-i and results are shown (n = 3, mean ±SD). G. Reduction in the concentration of AcCoA in H37Rv-infected cells after treatment with UK5099 (5 µM) [59]. AcCoA levels were measured by LC-MS/MS in cell extracts generated at 36 hr p-i. (n = 3,mean ±SD, **p<0.01) H. Prevention of mitochondrial Pyr transport with UK5099 inhibits *de novo* lipid synthesis (FA), 3HB generation, necrosis (Nec), and LB accumulation (LB) in virMtb-infected cells. Data shown are for H37Rv-infected cells and FA, LB and 3HB levels were determined at 48 hr p-i., whereas necrosis was quantified at 72 hr p-i. Similar results were also obtained in THP-1 cells infected with either JAL2287 or BND433. Values (n = 3, ±S.D.) are expressed as the percent reduction in comparison with the corresponding levels in infected, but untreated, cells.

Key to the intracellular survival of Mtb is its ability to induce the host macrophage to differentiate into FMs [7], [13], [24]–[26]. This occurs through the accumulation of lipid bodies (LBs) in the cell, which are composed of triglycerides (TAGs) and cholesteryl esters (CEs)[27]. LBs function by serving as a nutrient source, and also by providing a secure niche, for the intracellular bacteria [25]–[27]. Therefore, it seemed probable that the *de novo* lipid synthesis induced by virMtb represented a precursor step for LB biogenesis. We verified this by treating virMtb-infected cells with either Atorvastatin [28] to suppress CL biosynthesis via HMGCoA reductase inhibition, or with the FA synthase (FAS) inhibitor C-75 [29]. Either of these inhibitors reduced the LB content of the host cell although the effect of C-75 was more pronounced (Figure 7A). Significantly, the intracellular bacillary load was also reduced (Figure 7B). We obtained similar effects upon silencing of the corresponding target enzymes by siRNA (Figure S5C and S5D), which confirmed that compromised mycobacterial survival was a result of suppression of host rather than bacterial lipid biosynthetic pathways. In this connection, an earlier study has reported upregulation of transcripts coding for ATP citrate lyase and HMGCoA reductase in human TB granulomas [30]. This observation would support the physiological relevance of our findings.

Figure 7C superimposes the time course profiles for 3HB and lipid (FA plus CL) synthesis by the virMtb-infected cells. Although the magnitude and kinetics vary, it is evident that the concomitant induction of both lipids and 3HB by the host cell constitutes a recurring theme of virMtb infections. We previously showed that 3HB also played a critical role in FM differentiation [13]. It acted by stimulating the antilipolytic receptor GPR109A to inhibit TAG turnover and, thereby, promoted LB accumulation [13]. Thus the complementary capabilities of provoking host cell lipid synthesis on the one hand, while simultaneously also suppressing TAG turnover on the other, likely enables Mtb to efficiently steer FM differentiation.

### Mtb-induced FMs are primed for necrosis

TAG accumulation in macrophages exhibit cytotoxicity, causing the cells to undergo necrosis [13], [31], [32]. Therefore, FM differentiation could also potentially account for the necrotic death of virMtb-infected macrophages. To test this we induced FM differentiation in THP-1 cells by stimulating them with 3HB. The consequent enhancement in TAG/LB levels also led to an increase in the population of necrotic cells (Figure 7D). Suppression of either TAG synthesis with Triacin C [33], or of LB biogenesis with CI-976 [13], however, inhibited this process (Figure 7D). Similarly, H37Ra-infected cells could also be induced to switch from apoptotic to necrotic death in the presence of exogenous 3HB. This switch was sensitive to the GPR109A inhibitor Mepenzolate bromide (MPN)[13] (Figure 7E), supporting a mediatory role for LBs.

On the converse side, necrosis of virMtb-infected cells was suppressed by inhibition of pathways mediating TAG accumulation in macrophages (Figure 7F). Pathways that were inhibited included citrate export from mitochondria, its subsequent catabolism, lipid synthesis, and TAG turnover (Figure 7F). Further, suppression of mitochondrial AcCoA generation (Figure 7G) by addition of the mitochondrial pyruvate carrier (MPC) inhibitor UK5099 [34], [35] resulted in concomitant inhibition of both FA and 3HB synthesis by the host cell (Figure 7H). Consequently, LB formation was also inhibited and the virMtb-laden cells were protected from necrotic death (Figure 7H). RNAi-mediated silencing of the human *MPC1* and *MPC2* genes also produced similar effects (Figure S5E; S5F and S5G), confirming the target specificity of the effects of UK5099. These collective results, therefore, establish that TAG cytotoxicity prevails in infected macrophages, and that initiation of FM differentiation in virMtb-infected cells also constitutes commitment to the eventual death by necrosis.

### Metabolic thresholds, bacillary load, and pathogenesis

**Figure 8:**
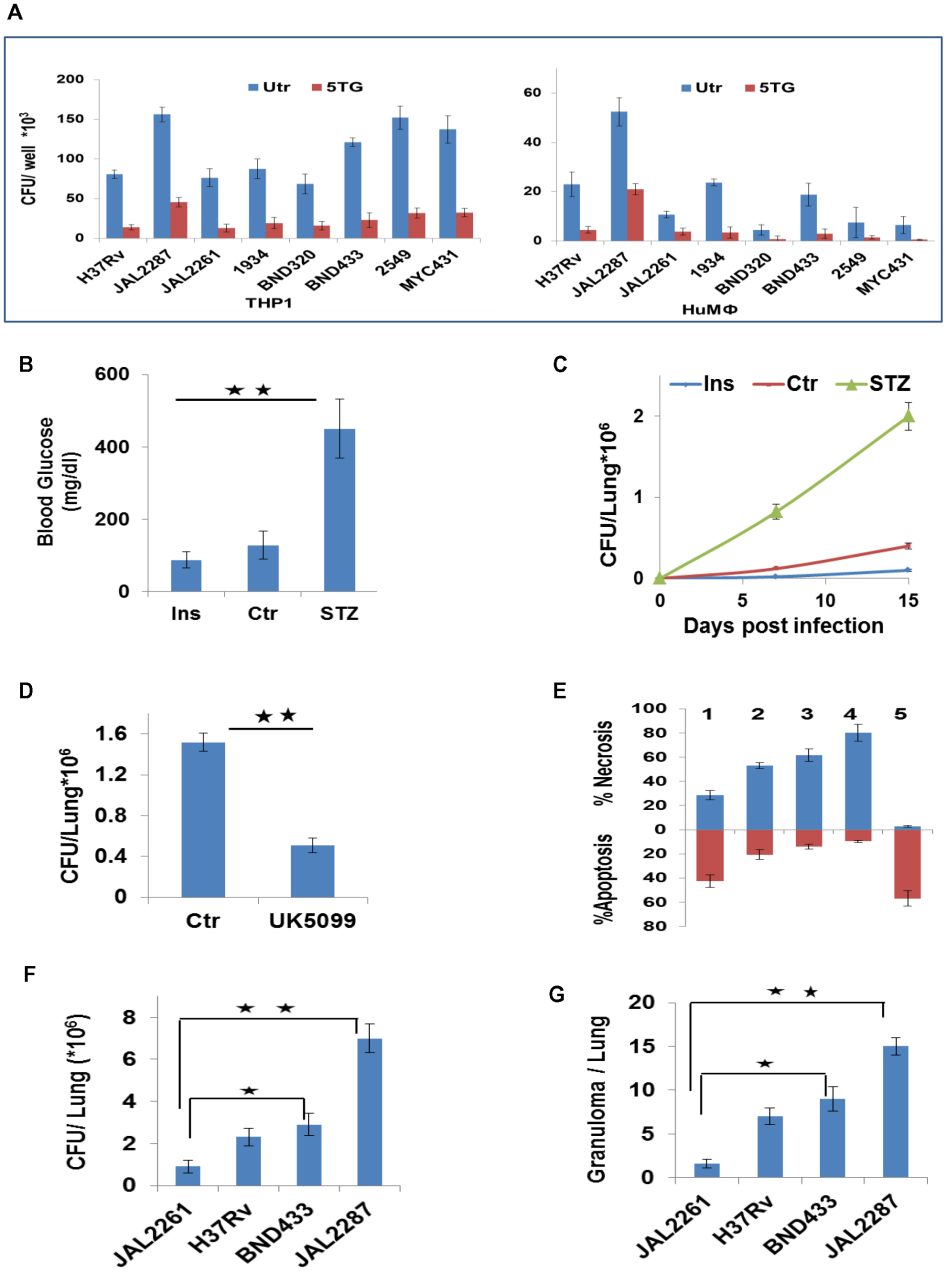
Metabolic thresholds influence Mtb pathogenesis. A. In parallel sets, THP-1 cells and primary human PBMC-derived macrophages (HuMφ were infected with the indicated strains, and cultured either in complete RPMI (Utr), or in media supplemented with 5TG (10 mM). Bacterial load was then determined as CFU values at 72 hr p-i (n = 3, mean ±SD). B, C. Groups of 6–8 mice were either left untreated (Ctrl), or rendered hypoglycemic through insulin administration (Ins, 60 IU/kg/day), or were treated with Streptozotocin (180 mg/kg) to induce diabetes (STZ). Panel ‘B’ gives the blood sugar levels (mean ±S.D.) in the mice after the respective treatments. These mice were infected with H37Rv and lung CFU values determined at the times indicated (C). Values are the mean ±S.D. (significance *p<0.05, **p<0.01). D. Lung CFUs in H37Rv-infected diabetic mice that were either left untreated (Ctr), or treated with 12 mg/Kg/day of UK5099 (8 mice/group, mean ±SD, significance *p<0.05). E. The relative proportion of cells undergoing either apoptosis (brown bars) or necrosis (blue bars) in THP-1 cells infected with the individual virMtb strains is shown. Result for a parallel set of JAL2287-infected cells that were maintained in medium supplemented with 5TG is also given. Analysis was at 72 hr p-i (n = 3, mean ±SD). 1: JAL2261; 2: H37Rv; 3: BND433; 4: JAL2287; 5: JAL2287 + 5TG. F–G. Lung CFUs in mice after 30 days of infection with the indicated Mtb strains (F). Data are mean ± S.D. of 8 mice/group. Panel ‘G’ gives the total number of granulomas seen in three lung sections from the mice in each group. *p<0.05, **p<0.01.

The mode of death adopted by infected macrophages is relevant to the pathophysiology of Mtb infection. While apoptosis constitutes a bactericidal response that limits disease, necrotic death facilitates escape of bacilli from the host cell to promote the spread of infection [36]–[41]. Our finding that extracellular glucose concentrations influence the extent of apoptosis of Mtb-infected macrophages would then also link glucose availability to the control of infection. This relationship could be demonstrated in both THP-1 and HuMΦ cells, in the context of infection with diverse clinical isolates (Figure 8A). Further, in mouse models for diabetes and hypoglycemia, the glycemic status clearly impacted on Mtb growth (Figure 8B&C). Importantly here, the relatively enhanced bacterial growth in diabetic mice could be suppressed by UK5099 treatment (Figure 8D), although this compound had no effect on extracellular cultures of Mtb or on macrophage viability (Figure S6A and S6D). These results therefore underscore the dependence of *in vivo* Mtb growth on host cellular metabolic flux.

The capacity of the infecting strain to enhance glucose uptake by the host macrophage (Figure 3A–C) could also additionally superimpose on the constraints of glucose availability, and contribute towards determining survival versus death of the infected macrophage. To investigate this we took another Mtb isolate, JAL2261, which was compromised in its ability to potentiate glucose uptake rates (<25% effect) in both THP-1 and HuMΦ cells (Figure S6B). Its inclusion alongside JAL2287, BND433, and H37Rv therefore, expanded the quantitative range of perturbations in host cell glycolytic flux that could be studied. Notably, virMtb strain-dependent effects on macrophage glucose uptake did not strictly correlate with the corresponding intracellular bacillary density (Figure S6B), suggesting that the former property was at least not entirely dependent on the latter.

The magnitude of Mtb-elicited acceleration of glucose uptake by the host cell clearly influenced the mode of death that resulted. Apoptosis of the host cell predominated when the pathogen exerted only a marginal effect on glucose uptake (JAL2261, Figure 8E). However, as the capacity of the infecting strain to stimulate host cell glucose uptake increased, the death response progressively shifted towards necrosis (Figure 8E). 5TG addition neutralized this shift confirming its dependence on the glycolytic flux (Figure 8E). Consistent with these *ex vivo* observations, both the extent of bacterial growth (Figure 8F) and the magnitude of the granulomatous response (Figure 8G and Figure S6C) in mice also displayed a trend that correlated with the ‘strength’ of the infecting strain to initiate glycolysis in the host macrophage. Admittedly, however, it is presently difficult to distinguish the relative contribution of metabolic effects from that of other factors such as bacterial burden, to the observed pathology.

## Discussion

The ability to control and/or manipulate the timing and mode of death of infected cells plays an important role in mycobacterial infection [36]–[41]. Control of death by the host generally initiates apoptosis. This then functions as an innate defense mechanism that restricts microbial spread and enhances the induction of adaptive immunity. It is generally believed that virMtb inhibits apoptosis early in infection and, instead, induces necrosis of the host cell at later time points. This outcome provides for release of viable bacteria from the infected cells thereby promoting re-infection and, ultimately, transmission of disease. In addition, necrosis of infected macrophages has also been implicated in granuloma formation and inflammatory tissue damage [30], [42]. Our present study substantially enriches this general view by elucidating the scheme employed by virMtb to regulate death pathways of the host macrophage. Significant in this context is the identification that this process was controlled at the level of host cell metabolism, with regulation also impacting on pathology of the disease.

Resolution of the pathogenetic strategies employed by Mtb was aided by our study design, which specifically incorporated several distinctive features to enable this end. First was the inclusion of a mycobacterial panel that collectively presented a manifold of virulence traits. By complementing this with a time-series analytical protocol, we could distinguish between the macrophage metabolic response profiles that correlated with either the elimination of non-pathogenic bacteria, the curtailment of an attenuated strain, or even with the successful cooption of host cell function by virulent bacilli. Interrogation of the data with ODE models then helped identification of the key interference points that dictated expression of Mtb virulence.

Our discovery that the capacity to modulate glycolytic flux of the host macrophage dictated Mtb pathogenicity provides a new perspective on host-pathogen crosstalk. As demonstrated, this ability facilitated pathogen survival by subverting apoptosis of the infected cell. Subsequent studies clarifying the sole dependence of macrophages on glycolysis for their bioenergetic needs then revealed the basis for this effect. Thus, modulation in glycolytic flux was alone sufficient to determine the balance between survival and apoptotic death of the host cell. Our ODE models subsequently identified that Mtb virulence in fact derived from the targeted perturbation of only two steps in the pathway. One of these involved augmentation of glucose uptake by the macrophage, while the other fostered this effect by increasing substrate availability for the payoff phase of glycolysis. As a result, the rate of ATP synthesis was markedly enhanced in virMtb-infected cells. Thus, whereas the default consequence of infection on the host macrophage was to impose an energy demand that breached the homeostatic barrier, manipulation of ATP synthesis by virMtb circumvented this to ensure that infected cells remained viable.

In conjunction with suppression of host macrophage apoptosis, establishment of infection also requires that the pathogen tend to its own survival needs within the intracellular milieu [43]. Here again our study yielded important insights by delineating how the glycolysis-mediated inhibition of apoptosis was allied with downstream regulation of select mitochondrial pathways to drive FM differentiation. The LBs generated as a result are known to provide a privileged niche for the intracellular bacteria [2], [13], [25], [26], [30]. As shown, the proficiency of pathogen in this regard stemmed from its ability to stimulate the complementary host processes of *de novo* lipid synthesis to generate TAGs on the one hand, and production of 3HB to inhibit lipolysis on the other. The concerted functioning of these two pathways was confirmed by the fact that pharmacological inhibition of either one of them compromised FM differentiation of virMtb-infected cells. Thus, regulation of LB biogenesis constitutes an indispensable ingredient of Mtb pathogenicity.

While FM differentiation facilitated stabilization of the infection, we also found that continued accumulation of TAG eventually led to necrotic death of the cells. That is, the control over macrophage metabolism by pathogen persisted up to the point of securing a mechanism for eventual escape of the bacteria from these cells. These latter findings add to the earlier results and highlight the fluidity with which the individual stages that contribute to pathogenesis are integrated, and governed, by the pathogen. Noteworthy here was that this was achieved through a parsimonious strategy of targeting only select reactions. In this connection we note that both the temporal and quantitative aspects of virMtb-induced host cell lipid synthesis varied significantly between the individual strains. Therefore, it will be interesting to determine if the end point of TAG-induced necrosis is also differentially regulated depending on the Mtb strain and/or the bacillary load.

Diabetes is an established risk factor for TB [44]–[46], and at least one causal factor suggested is the compromised immunity of hyperglycemic individuals [47], [48]. Our present finding that circulating glucose levels also contribute to pathogenicity by influencing macrophage apoptosis, however, reveals an additional facet to this relationship. That is, the glycemic status of an individual also impacts on the innate defense mechanism that controls Mtb infection. Notably, there is emerging evidence to suggest that the link between glucose availability and fate of the infected macrophage has broader implications that extend beyond the case of Mtb infection alone. Thus, for example, it may explain earlier findings that glucose availability also sustains chronic *Brucella abortus* infection in macrophages [49]. Similarly, it may also rationalize the recent discovery that control of glucose levels in the airway surface liquid serves as a mechanism for maintaining sterility of the lung airways [50].

Our observation that virMtb strains differ in their capacity to promote uptake of glucose by the host macrophage is also pertinent as it adds a complimentary dimension to the issue of glucose-dependency of Mtb infection. As shown, this property syndicates glucose availability with the cellular glycolytic rate by scaling the rate of glucose uptake by the host cell. Thus, glycemic levels and macrophage glycolysis together constitute an axis that integrates properties derived from both host and pathogen, in deciding the course of infection. That is, the ensuing pathology in any Mtb-infected individual is likely a result of the integrated effects of the individual's glycemic status, and nature of the infecting strain. Further analysis of this relationship may provide insights into factors that determine susceptibility to TB. Additionally, it may also be worthwhile to explore whether necrosis of Mtb-laden FMs contribute to the observed link between TB and atherosclerosis, with the consequent risk potentiation of ischemic stroke [51]–[55].

The facility with which Mtb continues to persist and disseminate in the human population remains a daunting challenge [6]. Our predominant focus on host immunity as a determinant of disease susceptibility is yet to yield results in terms of an effective vaccine against TB, or even a less ambiguous description of the correlates of protection. A case may, therefore, be made for revisiting the existing paradigms for understanding Mtb pathogenicity. By highlighting the extent to which metabolic intervention dictates the pathology of TB, our present report would indeed support such a view. Significant in this context is our identification that chokepoint interactions do exist that may be indifferent to either genotype or phenotype of the Mtb strain. Such a scenario then, also raises the possibility of exploiting such interactions for the development of new therapeutic strategies.

## Methods

### Cell culture, infection and CFU determination

PMA-differentiated THP-1 cells (routinely tested to be Mycoplasma free) were infected with mycobacteria at a multiplicity of infection of 10 over a 4 hr period. This was followed by amikacin treatment for 2 hr to remove any extracellular bacteria as described earlier [13], [17]. Human PBMC-derived monocytes were isolated by centrifugation over Ficoll-Paque from heparinized blood and then selected by adherence. Monocytes were then spontaneously differentiated into macrophages as described in Methods S1. For determination of CFUs, infected cells were lysed in 0.06% SDS and then plated on 7H11 agar plates supplemented with OADC and 5% glycerol. The Mycobacterial strains used in this study have been described earlier [13], [17].

### Ethics statement

The animal care and use protocol was reviewed and approved by the Institutional Animal Ethics Committee (IAEC) of International Centre of Genetic Engineering and Biotechnology, New Delhi (ICGEB, New Delhi). The animal care and use protocol adhered to the Committee for the Purpose of Control and Supervision of Experiments on Animals (CPSEA), Government of India, guidelines for Laboratory animal Facility. The reference number for the approval (IAEC number) is ICGEB/AH/2013/1/IMM32.

### Inhibitor treatment

Inhibitor treatment of infected and uninfected controls was initiated after Amikacin removal post infection (6 hrs p-i.). The medium containing the appropriate dose of inhibitor was replenished at 36 hrs and where needed, at 64 hrs p-i. The inhibitors were used at the following final concentrations: Atorvastatin at 10 µM, C75 at 20 µM, UK5099 at 5 µM, Rotenone at 1 µM; Mepenzolate (MPN) at 100 nM; 3BP at 50 µM; Citrate Lyase inhibitor-3,5 dichloro-2-hydroxy-N-(4-methoxy biphenyl-3-yl) benzene sulphonamide (DCBS) at 50 µM; 1,2,3 benzene tricarboxylate at 200 µM; 5TG at 20 mM; 2DG at 12 mM; and Triacin C at 5 µM.

The above inhibitor concentrations were determined by generating dose response curves on H37Rv-infected THP-1 and HuMФ cells following the protocol described above. The concentration showing maximal efficacy, but without any cytotoxic effect on the host cells (as determined in parallel experiments of inhibitor to UI cells), was selected. Inhibitor cytotoxicity was determined by the MTT assay. Importantly, we also confirmed that – at the concentrations tested – none of the inhibitors employed had any effect on growth of cell-free bacterial cultures. (Figure S6A and S6D)

### Labeling of cells with ^13^C_6_-glucose and metabolite extraction

Labeled RPMI medium was prepared by adding 2 g/liter of ^13^C_6_-glucose or 0.3 g/liter of ^13^C_5_-glutamine to glucose or glutamine free RPMI media respectively followed by sterile filtration. For labeling, 5*10^6^ differentiated THP1 cells were switched to fresh unlabeled media 1 hour before labeling so as to reduce perturbations in metabolite levels at the time of the labeling [56]. Unlabeled media was then completely removed, cells were washed once with glucose free RPMI, and fresh ^13^C_6_-glucose or ^13^C_5_-glutamine labeled media supplemented with 10% dialyzed fetal calf serum was added for the desired times. Metabolic reactions were quenched by adding chilled (−75°C) mixture of methanol:water (80∶20, v/v) After incubation at 75°C for 10 minutes the cells were scrapped from the culture dish (kept on dry ice) and collected. The scrapped cell suspension was then vortexed, followed by centrifugation at 6000 g for 5 minutes at 4°C, and the supernatant was stored. The pellet was re-extracted two more times with 80% methanol at −80°C. The three extractions were pooled, centrifuged at 13000 g for 5 minutes to remove any debris, and dried under a Nitrogen stream. The dried samples were re-suspended in MS-grade water, and centrifuged at 13000 g, 4°C for 10 minutes. Supernatants were used for the LC-MS/MS analysis.

Metabolites were quantified by employing concentration-dependent standard curves for each metabolite as described in Methods S1. Mitochondrial and cytoplasmic fractions were generated as detailed in Methods S1 and the kinetic labeling data was similarly obtained. For determining the rate of lipid and cholesterol synthesis, cells were incubated in ^13^C_6_-glucose containing RPMI for 4 hours and total cellular lipids were extracted. Cellular TAGs were then hydrolyzed with HCl/CH_3_CN (1∶4, v/v) and Palmitic acid was analyzed by direct infusion on an Applied Biosystems/MDS Sciex Q 4000 TRAP linear ion trap mass spectrometer in a negative polarity mode. Determination of free cholesterol was achieved by converting it to cholesterol-3-sulfate, and then analyzing by mass spectrometry with the sulfate group providing the negative charge. The detailed protocol is provided in Methods S1.

### Glucose uptake assay

Cells were initially washed with glucose free RPMI followed by addition of glucose free RPMI supplemented with 200 µM 2-NBDG for 0,5,10,20 and 30 minutes. Subsequently, the labeling media was removed cells were washed in glucose free RPMI and lysed in 200 µl of a non-interfering buffer (1% Nonidet P-40, 1% sodium deoxycholate, 40 mM KCl, 20 mM tris pH7.4). The lysate was centrifuged at 13000 g at 4°C for 5 minutes to remove debris. Fluorescence of the internalized glucose was measured on a flourimeter at 535 nm (excitation wavelength 485 nm).

### TAG quantitation

TAG levels were measured after solubilizing cells in 5% (v/v) Triton X-100 with the Triglyceride Quantification Kit (Abcam), and following the instructions of the manufacturer.

### Cell death determination assay

To determine the mode of death, the Apoptosis/Necrosis Detection kit (Enzo) was used. At the time of assay the cells were fixed in 2% PFA and the protocol recommended by the manufacturer was followed. Cells were analyzed by confocal microscopy.

### Confocal microscopy

Cells were stained with the required reagents as mentioned in Methods S1. Stained cells were observed with a Nikon EclipseTi-E laser scanning confocal microscope equipped with 60X/1.4NA Plan Apochromatic DIC objective lens. DAPI and Lipid Tox/MitoSOX were excited at 488 nm, 408 nm and 543 nm with an argon ion, blue diode and a Helium-Neon laser respectively. The emissions were recorded through emission filters set at 515/30; 450 and 605/75. Images were acquired with a scanning mode format of 512×512 pixels. The transmission and detector gains were set to achieve best signal to noise ratios and the laser powers were tuned to limit bleaching fluorescence. The refractive index of the immersion oil used was 1.515 (Nikon). All settings were rigorously maintained for all the experiments.

#### Image analysis

All images were quantified using Image-Pro Plus version 6.0, a commercially available software package from Media Cybernetics. The mean fluorescence intensity per cell was determined with the help of density sum and area tools in the software. Density sum refers to the sum of intensity values of all the pixels of a counted spot. After obtaining mean fluorescence intensity per cell, the values were averaged over a minimum of 200 cells from three independent experiments. Values when depicted as fold increase or percent reduction refer to the increase over uninfected or in certain cases untreated (or control) sets.

### Animal experiments

Female BALB/c mice, 4–6 weeks of age (8 per group) were infected with H37Rv through aerosol exposure by delivering, during 30 min of exposure, between 100–150 bacteria per lung. The latter was determined by the culture of lung homogenates at 24 hr later. STZ (180 mg/kg) treatment was initiated by administering intra peritoneal injection once, 7 days before aerosol exposure.

In the appropriate groups, bovine insulin (60 IU/kg/day) was administered via a mini osmotic pump (Alzet), implanted subcutaneously on the back of mice. Insulin treatment was started 24 hrs prior to aerosol administration. The mice in the corresponding control group were similarly implanted with saline filled mini osmotic pumps.

In a separate experiment female BALB/c mice, 4–6 weeks of age (8 per group) were treated with UK5099 (12 mg/kg/day), via a mini osmotic pump implanted subcutaneously on the back of mice, 24 hrs prior to aerosol infection with JAL2287 strain of Mtb. Blood sugar levels for all treated mice and controls were monitored every third day. At the appropriate times, these mice were sacrificed and mycobacterial load in lung was monitored. In all animal studies, the author taking CFU counts was not aware of the sample identity, the nature of treatment and thus the counts were taken on an unbiased background.

### Statistical analysis

We determined the statistical significance by using the unpaired two-tailed Student's t test with origin software. p<0.05 was taken as statistically significant.

### Mathematical modeling

#### Determination of saturation concentrations

Saturation concentration (stability point) of a ^13^C-labeled incorporated metabolite depicts the point of ^13^C-labeled concentration beyond which no significant change in the concentration was observed during the 60 minutes labeling period. This was identified for each of the metabolites by a modified 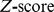 method using the formula, 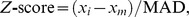
where, ‘*x_i_’* is the Concentration of ^13^C-labeled metabolites, ‘*x_m_*’ is the median of the concentration of ^13^C-labeled metabolites and ‘MAD’ is the median absolute deviation obtained by using the formula,

MAD  =  median{abs(*x_i_* - *x_m_*)}.

#### Determination of rates of reactions

Net rate of incorporation of ^13^C-labeled into a metabolite was determined by plotting the concentration of ^13^C-labeled metabolite against the time taken for the label incorporation and then calculating the slope of the graph at half the ^13^C saturation concentration reached by the metabolite (linear part of the graph). We similarly obtained the rate of degradation of ^12^C isotope of metabolites except that the time taken to reach a half-minimal value considered.

#### Flux analysis and model formulation

The influx rate was obtained by respectively adding the experimentally calculated slope of ^12^C isotope degradation and slope of ^13^C-label incorporation into each of the metabolites. The slope of ^12^C isotope degradation was used as the outflux rate. Influx to a metabolite was mathematically represented as *x_i_ = a_i_+b_i_* and outflux from this metabolite as, *x_i_ = b_i_*, where *a_i_* was the slope of the kinetics of ^13^C –label incorporation and *x_i_* and *b_i_* the slope of the kinetics of consumption of the ^12^C isotope of the same metabolite *x_i_* (Figure S3E).

#### ODE model

A coupled ordinary differential equation model of CCM was constructed based on the pathway map shown in Figure 2A. The model consisted of 21 differential equations, written to maintain flux balance. Equations of the model described the rates of loss and the creation of particular labeled forms of metabolites after feeding of ^13^C-labeled glucose. The system of differential equations is detailed in Methods S1. The equations were written with an initial condition at origin (satisfying the experimental condition that ^13^C initially present in the metabolites was zero). In the equations, [*x_i_*] denotes the concentration of ‘*i* ’ metabolite with ^13^C carbon labeling; *r_i_* is the reaction rate of different metabolites; *k_i_* denotes the extra flux coming from outside to the considered metabolic network and *δ_i_* denotes the extra flux going out of the considered metabolic network.

#### Parameter estimation

We used the kinetics of ^13^C carbon-labeled nutrients into downstream metabolites to dissect metabolic fluxes. The influx in a particular metabolite was divided within the parameters *k_i_* and the incoming *r_i_*. Out-flux was divided within the parameters *δ_i_* and the outgoing *r_j_* (*i≠j*). Using the simple assumption that the consumption rate of a metabolite is equal to the synthesis rate of the subsequent metabolite we calculated the reaction rates (*r_i_*) between two consecutive metabolites present in the metabolic pathway by taking the minimum value between the out-flux rate of a metabolite and the influx rate of the next metabolite. The remaining rate was adjusted with the extra input *k_i_* and/or output terms *δ_i_*. The final flux analysis for uninfected cells is shown in Table S5.

The estimated parameters were then used to calculate the steady state concentration of the metabolites from the proposed model using ode23's inbuilt Matlab code, and compared with the experimentally obtained saturation concentration of ^13^C carbon-labeled metabolites. To satisfy the ^13^C-labeled metabolites saturation concentration (obtained experimentally), we varied different parameters keeping the input and output flux balanced. For example, the parameter set depicted in Table S5 was used to predict the steady state concentration of ^13^C-labeld metabolites for uninfected cell, given in Figure 2B. Model predictions (Tables S6 and S8) and validations (Tables S7 and S9) are described in detail in Methods S1.

### HPLC

High-pressure liquid chromatography (HPLC) was performed on an Agilent 1260 infinity Binary HPLC (Agilent technology, Waldbronn, Germany) equipped with a degasser, and an auto sampler. In all, three types of columns were used in the study- i) Aminopropyl, ii) C-18, iii) Cyano column. The auto sampler was maintained at 4°C to ensure sample stability. A flow rate of 200 µl/min and sample injection volume of 20 µl was maintained in the study. Elutes were continuously directed to the mass spectrometer with the help of turbo ion source.

Targeted metabolomic analysis of 25 hydrophilic metabolites was performed on the aminopropyl column using multiple reactions monitoring (MRM). Agilent Polaris 5 NH_2_ 2×150 mm column was used with a non-linear gradient of 85–0% B over 36 min (Table S1). Solvent A was 5% ACN/H_2_O containing 10 mM ammonium acetate and 10 mM ammonium hydroxide; pH 9.4 and solvent B was 100% ACN.

### Mass Spectrometry (MS)

The HPLC was coupled with a hybrid 4000 QTRAP (AB SCIEX, Foster City, CA, USA) with a Turbo V ESI ionization source interface, and a computer platform equipped with a Solution Analyst software version 1.5 (ABSciex, Foster City, CA, USA), which was used for data acquisition and processing as described in Methods S1. The mass spectrometric parameters for the precursor and product ions selected in MRM for metabolites under study and their corresponding parent and daughter ions parameters are depicted in Table S2. Standards were purchased from Sigma Aldrich and were used to generate the MRM profile and optimizing the source and compound parameters. Additional information on the validation method, the LC-gradient profile (Table S1, Figure S7), isotopomer analysis (Tables S2, S3, S4 and Figures S8, S9, S10) and chromatographic approaches employed for the various metabolites are described in Methods S1.

### Accession numbers

GAPDH (Glyceraldehyde-3-phosphate dehydrogenase): P04406; GLUT1 (Solute carrier family 2, facilitated glucose transporter member1): P11166; GLUT3 (Solute carrier family 2, facilitated glucose transporter member 3): P11169; HMGCR (3-Hydroxy-3-Methylglutaryl-CoA Reductase):P04035; FAS(Fatty Acid Synthase):P49327; GPR109A (Hydroxycarboxylic acid receptor 2):Q8TDS4; MPC1(Mitochondrial pyruvate carrier 1): Q9Y5U8; MPC2(Mitochondrial pyruvate carrier 2): O95563.

## Supporting Information

Figure S1 **Differential isotope labeling profiles of host versus bacterial metabolites.** Comparison of the labeling profile of host (blue line) and bacterial (red line) metabolites in H37Rv-infected cells. At 24 hr p-i., infected cells were pulsed with ^13^C_6_-glucose followed by host and bacterial metabolite isolation as described in Methods S1. Data is represents the percentage of ^13^C labeled metabolite pool in either fraction. (n = 3 mean ±SD).
(TIF)

Figure S2 **Model predicted concentrations for uninfected THP-1 cells, the experimental values and MRM transitions.** A. A comparison of the ^13^C-label incorporation rate, and the consumption rate of the corresponding ^12^C-labeled isotopomer in uninfected THP-1 cells (Experimental, n = 3 mean ±SD), with those that were fed into the model. B. MRM transitions used for identification of the individual metabolites by LC-MS/MS in our study. Abbreviations used are: Glucose 6-phosphate (G6P), Fructose bisphosphate (FBP), Dihydroxyacetone phosphate (DHAP), 3-phosphoglycerate (3PG), 2-phosphoglycerate (2PG), Phosphoenolpyruvate (PEP), Pyruvate (PYR), Lactate (LAC), Citrate (CIT), Aconitate (ACT), α-ketoglutarate (AKG), Succinate (SUC), Fumarate (FUM), Malate (MAL), Oxaloacetate (OA), Ribulose 5-phosphate (R 5-P), Phosphoribose diphosphate (PRD), Inosine monophosphate (IMP), Adenosine monophosphate (AMP), Nicotinamide adenine dinucleotide (NAD), Mevalonate (MVA), Guanosine monophosphate (GMP), AcetylcoA (AcCoA), malonylCoA (MaCoA), HMGCoA, 3-hydroxybutyric acid (3HB), Adenosine diphosphate (ADP), Adenosine triphosphate (ATP), and nicotinamideadenine dinucleotide (NADH).
(TIF)

Figure S3 **Glycolytic dependence of THP-1 macrophages and Mtb induced GLUT receptor upregulation.** A. Distribution of ATP levels between the cytoplasm and mitochondria of uninfected cells (n = 3, mean±SD, significance **p<0.01). The purity of the fractions was determined by a Western blot analysis of each fractions for the mitochondrial marker Cytochrome C oxidase (MTCO2), and the cytoplasmic marker GAPDH. ATP levels were determined by LC-MS/MS as described in the text. B-D. Effect of the inhibition of glycolysis on mitochondrial membrane potential by JC- 1 staining (B), ATP levels (C), and apoptosis (D) in UI cells (n = 3, mean ±SD, significance **p<0.01). E. Methodology adopted for estimating metabolite steady state concentrations from the corresponding synthesis and consumption rates (see Methods for details).
(TIF)

Figure S4 **Phenotypic properties of the mycobacterial strains and infection-induced effects on glucose transporters.** A. PMA-differentiated THP1 cells were infected with each of the mycobacterial strains at an MOI of 10∶1. Intracellular levels persisting at the indicated times was determined in terms of the colony forming units (CFU) present in the cell lysates. Values are the mean (±S.D.) of three separate experiments. B. Mtb virulence regulates mode of host cell death. Shown are the proportion of cells undergoing either apoptosis or necrosis and values represent an average of >100 cells (1:*M.smeg*, 2:H37Ra, 3:H37Rv, 4:JAL2287, 5:BND433). C. Mtb-infection induces GLUT1 and GLUT3 expression in PBMC-derived human macrophages. Values represent the fold change in mean fluorescence intensity (MFI) per cell in H37Rv-infected cells, over that in UI cells. Values are the mean ±SE obtained from at least 70 cells. D. Confocal microscopy images comparing GLUT-1 and GLUT-3 (green) expression in uninfected PBMC-derived human macrophages versus that in cells infected with H37Rv. DAPI (blue) was used for staining the nuclei.
(TIF)

Figure S5 **Model validation, lipid body accumulation, effect on bacterial CFU, and necrosis.** A. The panel on the left depicts the results of a simulation exercise that probed for the effects of varying glucose uptake rates on GP, FA, AcCoA and R5P synthesis. Simulation was done on the ODE model derived for JAL2287-infected cells, using the parameter values for the 24 hr infection time point. The panel on the right gives the corresponding, experimentally derived, results wherein JAL2287-infected cells (24 hr p-i) were treated with decreasing concentrations of glucose in the culture medium. Values are mean ±SE obtained from at least 3 independent experiments. A good correspondence in trends between the two sets of results is evident. B. Effect of glycolysis inhibition on apoptosis in infected and UI primary human monocytes. UI and H37Rv infected cells were either left untreated or were treated with increasing concentrations of 5TG. Cells were scored for percentage of apoptotic cells at 24 hours p-i. (n = 3, mean ±SD). C–D. RNAi-mediated silencing of *HMGCR* or *FAS* inhibits LB accumulation and CFU. (C) Results are shown as percent reduction in LB accumulation in H37Rv-infected cells, relative to that obtained in cells mock-transfected with GFP-specific siRNA. Data are from one of three independent experiments, and represent an average of 200 cells ±S.E. (D) The corresponding effect on bacterial CFU values, in terms of percent reduction from that in mock siRNA-transfected (GFP-specific) cells. The efficiency of silencing was determined by Western blotting for both the proteins after silencing (FASN; HMGCR). E–G. RNAi-mediated silencing of either *MPC1* or *MPC2* on E) LB accumulation and G) necrosis in H37Rv- infected cells. Data represent an average of 200 cells and are presented in terms of percent reduction relative to the corresponding value in GFP-silenced cells; n = 3, mean ±SD , **p<0.01). F) Representative confocal images obtained after Lipid Tox staining are also shown. The efficiency of silencing was determined by Western blotting using antibodies for the transporter proteins after silencing.
(TIF)

Figure S6 **Inhibitor treatment of cells and free bacterial cultures.** Glucose uptake efficiency and bacterial load. A. H37Rv was grown in liquid culture (7H9 medium) either in the absence or presence of the indicated drugs. At the indicated time points the bacterial growth was determined in terms of the O.D. values. (n = 3, mean ±S.D.). Inhibitors were used at the following concentrations: UK5099-5 µM; Atr-10 µM; C75- 20 µM; DCBS-50 µM; BTC-200 µM; MPN-100 nM, 3BP at 50 µM (n = 3 mean ±SE). B. Glucose uptake efficiency. The upper panel depicts the bacillary load per cell for individual strains obtained by 6 hours of infection. In the graph, the bars represent the percentage of total cell population harboring the indicated range of bacillary load (from 0 to >10 bacilli per cell). The Z axis represents the alteration in glucose uptake inflicted by the virulent strains compared to UI cells at 24 hr p-i. *Lower panel*: Infection with JAL2261 does not induce an increase in glucose uptake in either THP-1 or HuMФ cells. The rate of 2-NBDG uptake was monitored at 24 hours p-i. (n = 3 ,mean ±SD, *p>>0.05). C. Photomicrograph showing representative hematoxylin and eosin-stained lung sections of mice infected with H37Rv containing a small well defined granuloma (G) (left panel); and JAL2287 displaying two large granulomas (G) (right panel). D. Uninfected THP-1 and HuMФ cells were treated individually with UK5099 5 µM; Atr-10 µM; C75- 20 µM; DCBS-50 µM; BTC-200 µM; MPN at 100 nM, CI 976-1 µM; Triacin C at 5 µM; 3BP- 50 µM for 48 hrs and the percent viability over untreated cells was determined using the MTT assay (n = 5, mean ±S.D.).
(TIF)

Figure S7 **Extracted ion chromatography (XIC) of standards.** Chromatograms show the XIC of G6P, FBP, DHAP, 3PG/2PG, PEP, Pyruvic acid, CITRATE, Succinate, FUM, MALATE, OXA, R5P/Ribu5P, IMP, AMP, NAD, NADP, ADP, ATP, NADH and PRD as purified standards (1 µM) and their retention time as obtained after resolution on an Agilent Polaris 5 NH_2_ 2×150 mm column.
(TIF)

Figure S8 **XIC of G6P; 3PG/2PG; DHAP.** Figure depicting XIC (extracted ion chromatography) of G6P, transition 259→97, represent ^12^C and 265→97, represent ^13^C G6P; m/z 185→79, represent ^12^C and 188→79, represent ^13^C 3PG/2PG and m/z 169→97, represent ^12^C and 172→97, represent ^13^C DHAP.
(TIF)

Figure S9 **LC-MS/MS profile of cholesterol sulfate.** Representative LC-MS/MS results of co-eluted ^12^C and ^13^C carbon spectra for cholesterol sulfate (synthesized), which were identified by Luna CN column (100 Å, 2×150 mm 3 µ, Phenomenex, Torrance, CA, USA). ^12^C species is shown by m/z 465.7→97 and ^13^C by m/z 474.7→97 and 475.7→97.
(TIF)

Figure S10 **Ion spectra of free fatty acid obtained by direct infusion.** Representation of free fatty acids by the direct infusion method. A) standard palmitic acid spectra observed at m/z 255.4 in negative polarity. B) Representation of sample (Free fatty acids) spectra, ^12^C palmitic acid observed at m/z 255.4 and its respective ^13^C at m/z 267.4, 269.4 and 271.4 were convincingly identified and quantitated.
(TIF)

Methods S1 **The document provides detailed information on the materials used, the experimental protocols followed, the methodology optimized for LCMS/MS analysis of metabolite samples, and the mathematical model employed with its validations.**
(DOCX)

Table S1 **Gradient profile for LC-MS/MS method on Agilent Polaris 5NH_2_ 2×150 mm column.**
(DOCX)

Table S2 **Table represents metabolites, their fragment ion mass in negative polarity along with optimized compound dependent parameters used in MRM (multiple reaction monitoring).**
(DOCX)

Table S3 **Establishment of the LC-MS/MS method.** Table lists the retention time and linearity of metabolite standard calibration curves (R^2^) over the concentration range of standards used. Calibration curves were generated by injecting each standard five times over a concentration range of 500 nM-10 µM.
(DOCX)

Table S4 **Establishment of the LC-MS/MS method for cholesterol sulphate.** ^13^C and ^12^C transition and regression coefficient for cholesterol sulfate (Cholsul) are shown.
(DOCX)

Table S5 **The table shows flux distribution analysis from experimental ^13^C incorporation and ^12^C consumption rates for uninfected cells.**
(DOCX)

Table S6 **Table depicts the predicted glucose uptake rates as ratio over the corresponding rates obtained for uninfected cells.**
(DOCX)

Table S7 **Table showing experimentally determined glucose uptake rate as ratio with uninfected cells.**
(DOCX)

Table S8 **Model predicted rates of Fatty acid (FA) and Cholesterol (CL) synthesis in infected cells as ratio of UI cells.**
(DOCX)

Table S9 **Table showing experimentally determined fatty acid (FA) and cholesterol synthesis (CL) rates as ratios over UI cells.**
(DOCX)

Table S10 **Experimentally determined (Net rate) of ^13^C label incorporation into metabolites for THP1 cells infected with bacterial strains.**
(XLSX)
