# Supplementary figures and images for: Pathogenicity of Mycobacterium tuberculosis Is Expressed by Regulating Metabolic Thresholds of the Host Macrophage
Source: PLoS Pathog. 2014 Jul 24;10(7):e1004265. doi: 10.1371/journal.ppat.1004265 (PMC4110042; doi:10.1371/journal.ppat.1004265)

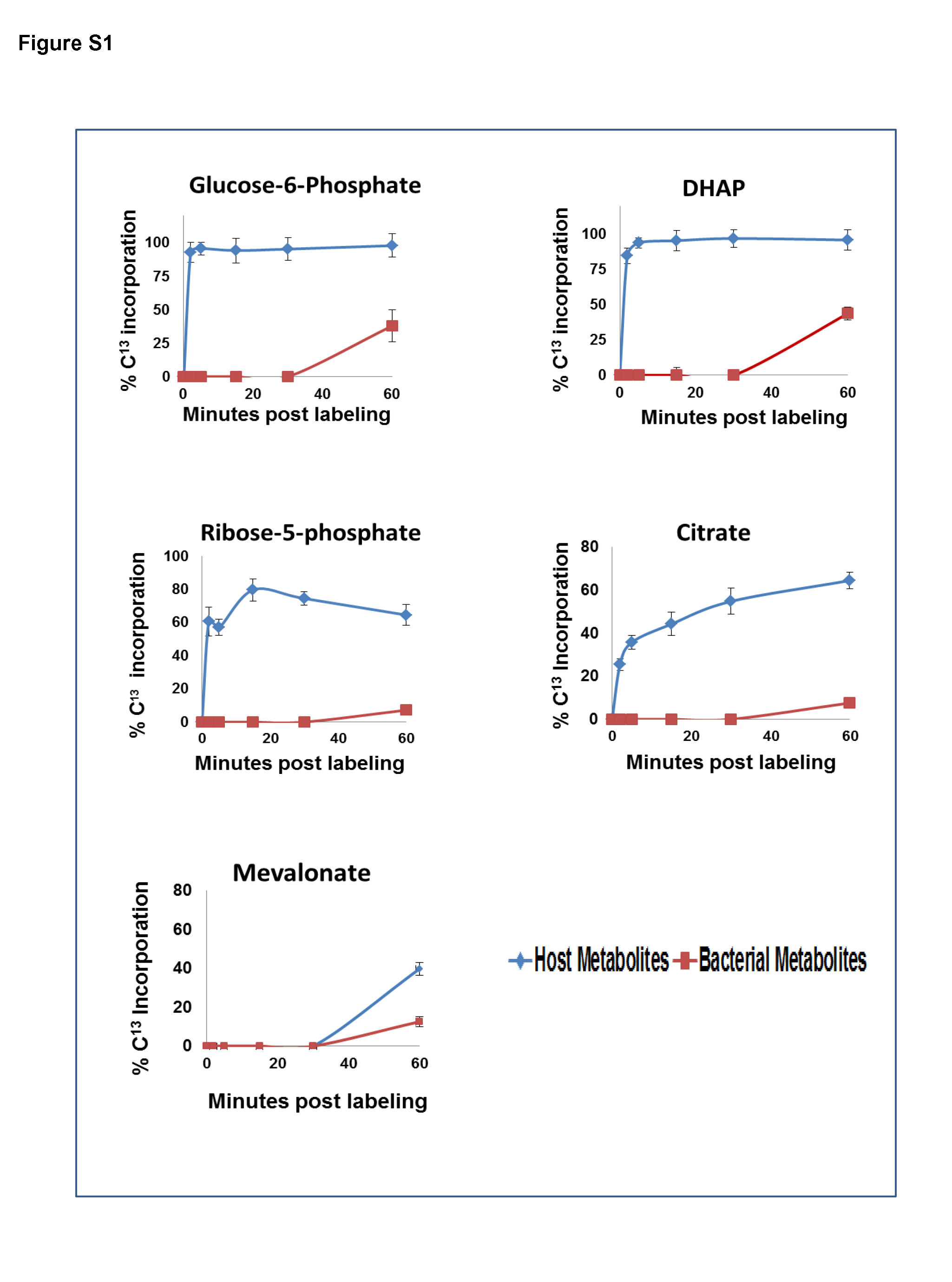

Supplement: Figure S1 — Differential isotope labeling profiles of host versus bacterial metabolites. Comparison of the labeling profile of host (blue line) and bacterial (red line) metabolites in H37Rv-infected cells. At 24 hr p-i., infected cells were pulsed with 13C6-glucose followed by host and bacterial metabolite isolation as described in Methods S1. Data is represents the percentage of 13C labeled metabolite pool in either fraction. (n = 3 mean ±SD). (TIF) [file ppat.1004265.s001.tif]

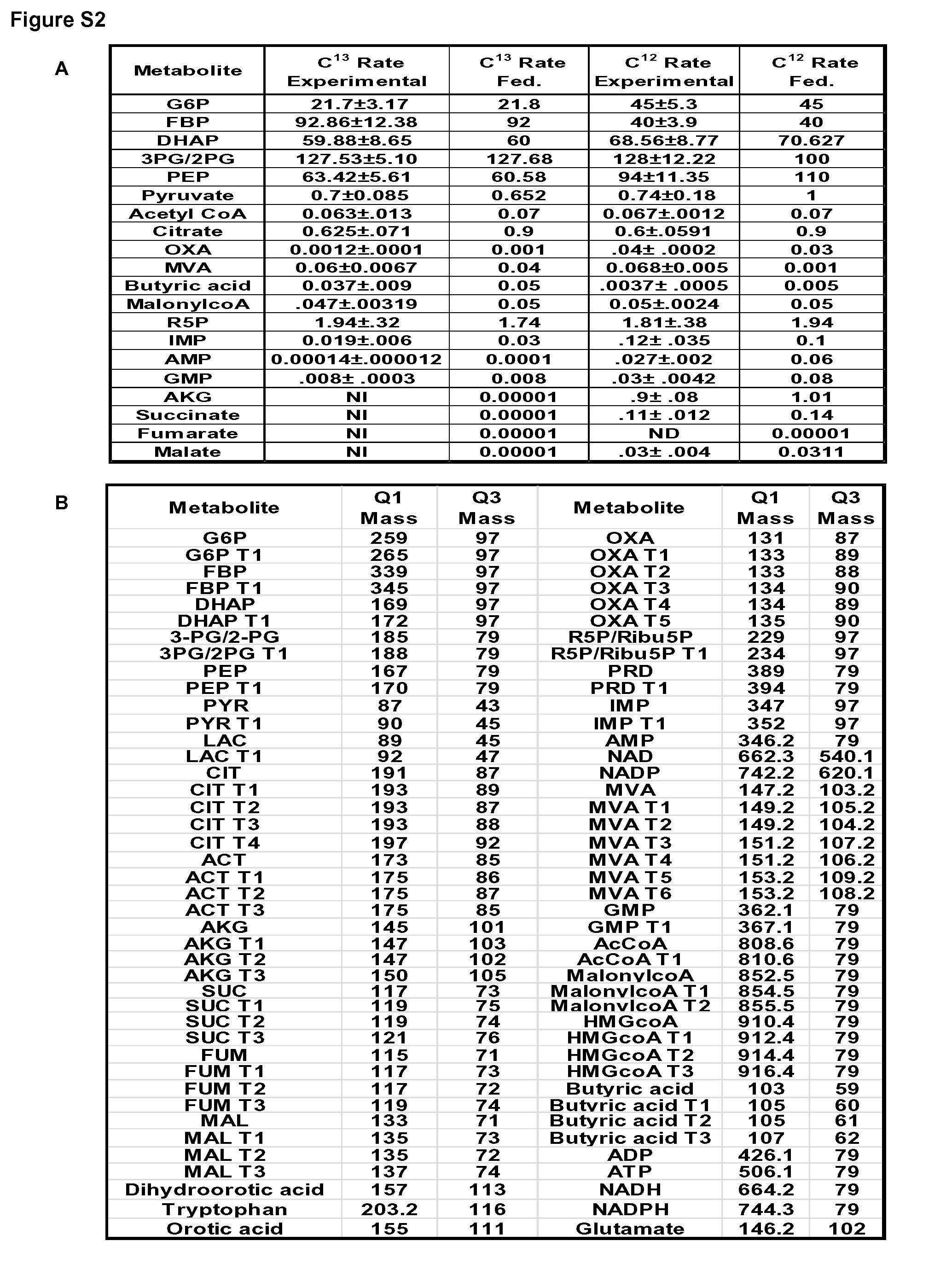

Supplement: Figure S2 — Model predicted concentrations for uninfected THP-1 cells, the experimental values and MRM transitions. A. A comparison of the 13C-label incorporation rate, and the consumption rate of the corresponding 12C-labeled isotopomer in uninfected THP-1 cells (Experimental, n = 3 mean ±SD), with those that were fed into the model. B. MRM transitions used for identification of the individual metabolites by LC-MS/MS in our study. Abbreviations used are: Glucose 6-phosphate (G6P), Fructose bisphosphate (FBP), Dihydroxyacetone phosphate (DHAP), 3-phosphoglycerate (3PG), 2-phosphoglycerate (2PG), Phosphoenolpyruvate (PEP), Pyruvate (PYR), Lactate (LAC), Citrate (CIT), Aconitate (ACT), α-ketoglutarate (AKG), Succinate (SUC), Fumarate (FUM), Malate (MAL), Oxaloacetate (OA), Ribulose 5-phosphate (R 5-P), Phosphoribose diphosphate (PRD), Inosine monophosphate (IMP), Adenosine monophosphate (AMP), Nicotinamide adenine dinucleotide (NAD), Mevalonate (MVA), Guanosine monophosphate (GMP), AcetylcoA (AcCoA), malonylCoA (MaCoA), HMGCoA, 3-hydroxybutyric acid (3HB), Adenosine diphosphate (ADP), Adenosine triphosphate (ATP), and nicotinamideadenine dinucleotide (NADH). (TIF) [file ppat.1004265.s002.tif]

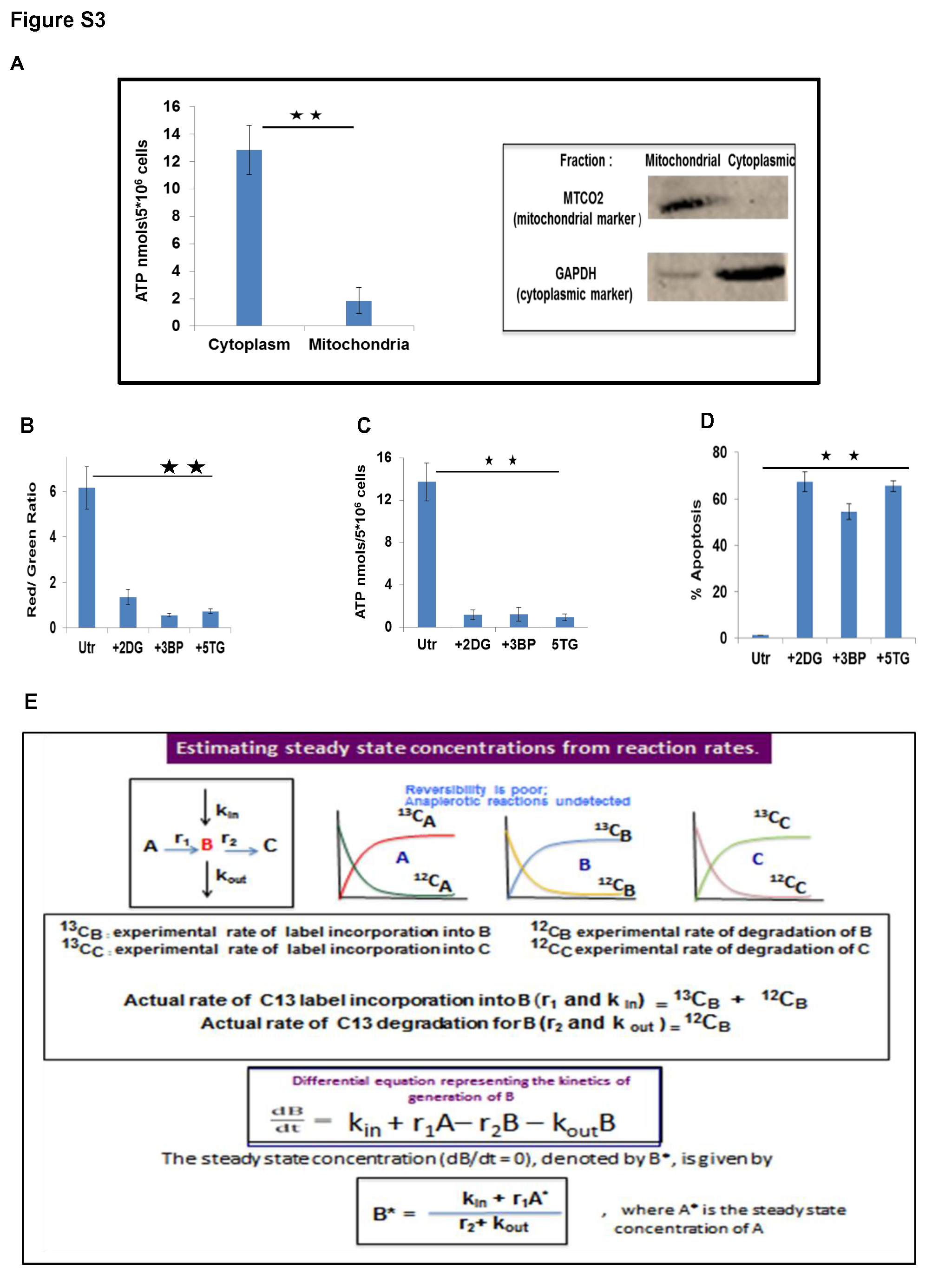

Supplement: Figure S3 — Glycolytic dependence of THP-1 macrophages and Mtb induced GLUT receptor upregulation. A. Distribution of ATP levels between the cytoplasm and mitochondria of uninfected cells (n = 3, mean±SD, significance **p<0.01). The purity of the fractions was determined by a Western blot analysis of each fractions for the mitochondrial marker Cytochrome C oxidase (MTCO2), and the cytoplasmic marker GAPDH. ATP levels were determined by LC-MS/MS as described in the text. B-D. Effect of the inhibition of glycolysis on mitochondrial membrane potential by JC- 1 staining (B), ATP levels (C), and apoptosis (D) in UI cells (n = 3, mean ±SD, significance **p<0.01). E. Methodology adopted for estimating metabolite steady state concentrations from the corresponding synthesis and consumption rates (see Methods for details). (TIF) [file ppat.1004265.s003.tif]

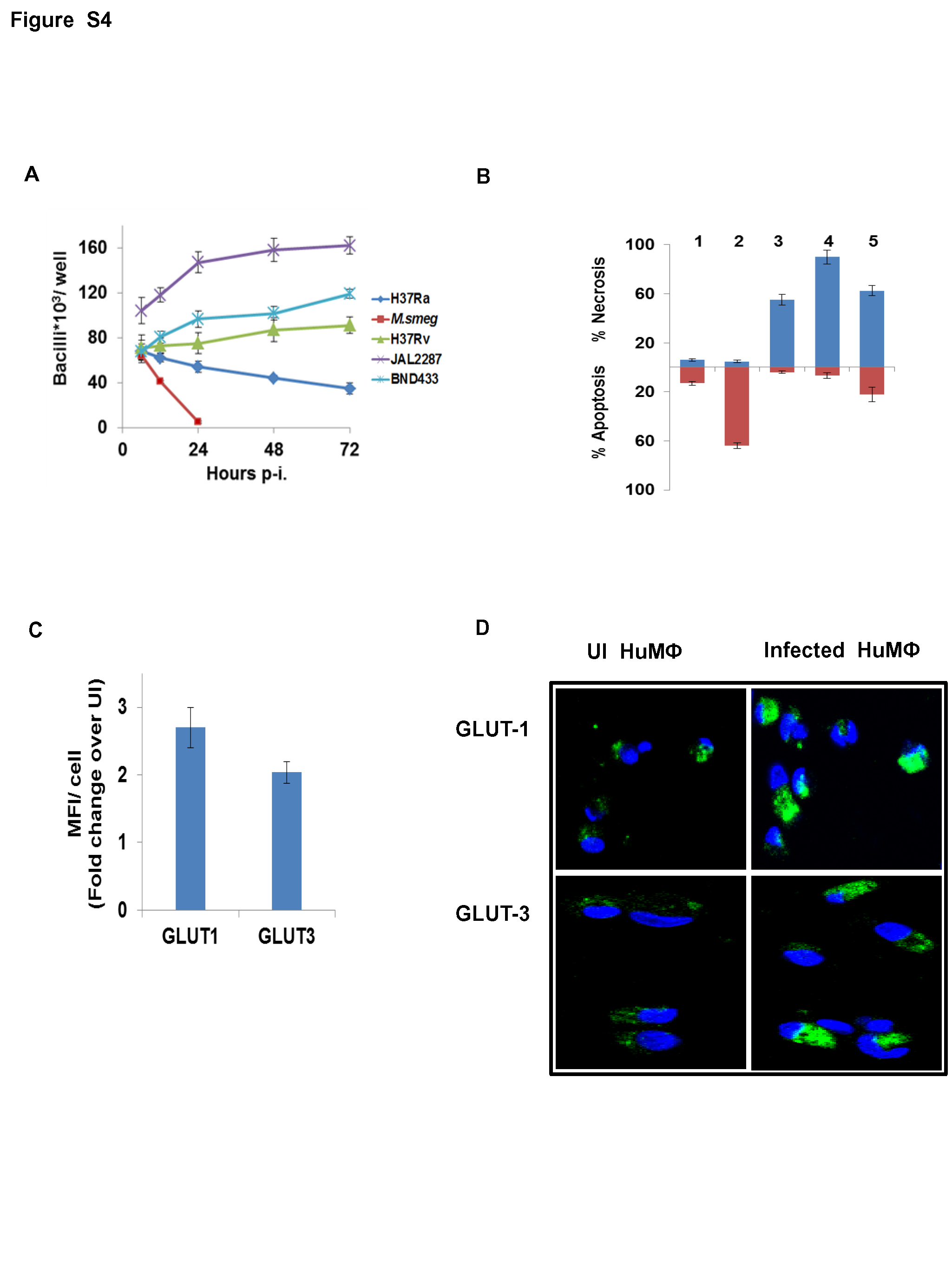

Supplement: Figure S4 — Phenotypic properties of the mycobacterial strains and infection-induced effects on glucose transporters. A. PMA-differentiated THP1 cells were infected with each of the mycobacterial strains at an MOI of 10∶1. Intracellular levels persisting at the indicated times was determined in terms of the colony forming units (CFU) present in the cell lysates. Values are the mean (±S.D.) of three separate experiments. B. Mtb virulence regulates mode of host cell death. Shown are the proportion of cells undergoing either apoptosis or necrosis and values represent an average of >100 cells (1:M.smeg, 2:H37Ra, 3:H37Rv, 4:JAL2287, 5:BND433). C. Mtb-infection induces GLUT1 and GLUT3 expression in PBMC-derived human macrophages. Values represent the fold change in mean fluorescence intensity (MFI) per cell in H37Rv-infected cells, over that in UI cells. Values are the mean ±SE obtained from at least 70 cells. D. Confocal microscopy images comparing GLUT-1 and GLUT-3 (green) expression in uninfected PBMC-derived human macrophages versus that in cells infected with H37Rv. DAPI (blue) was used for staining the nuclei. (TIF) [file ppat.1004265.s004.tif]

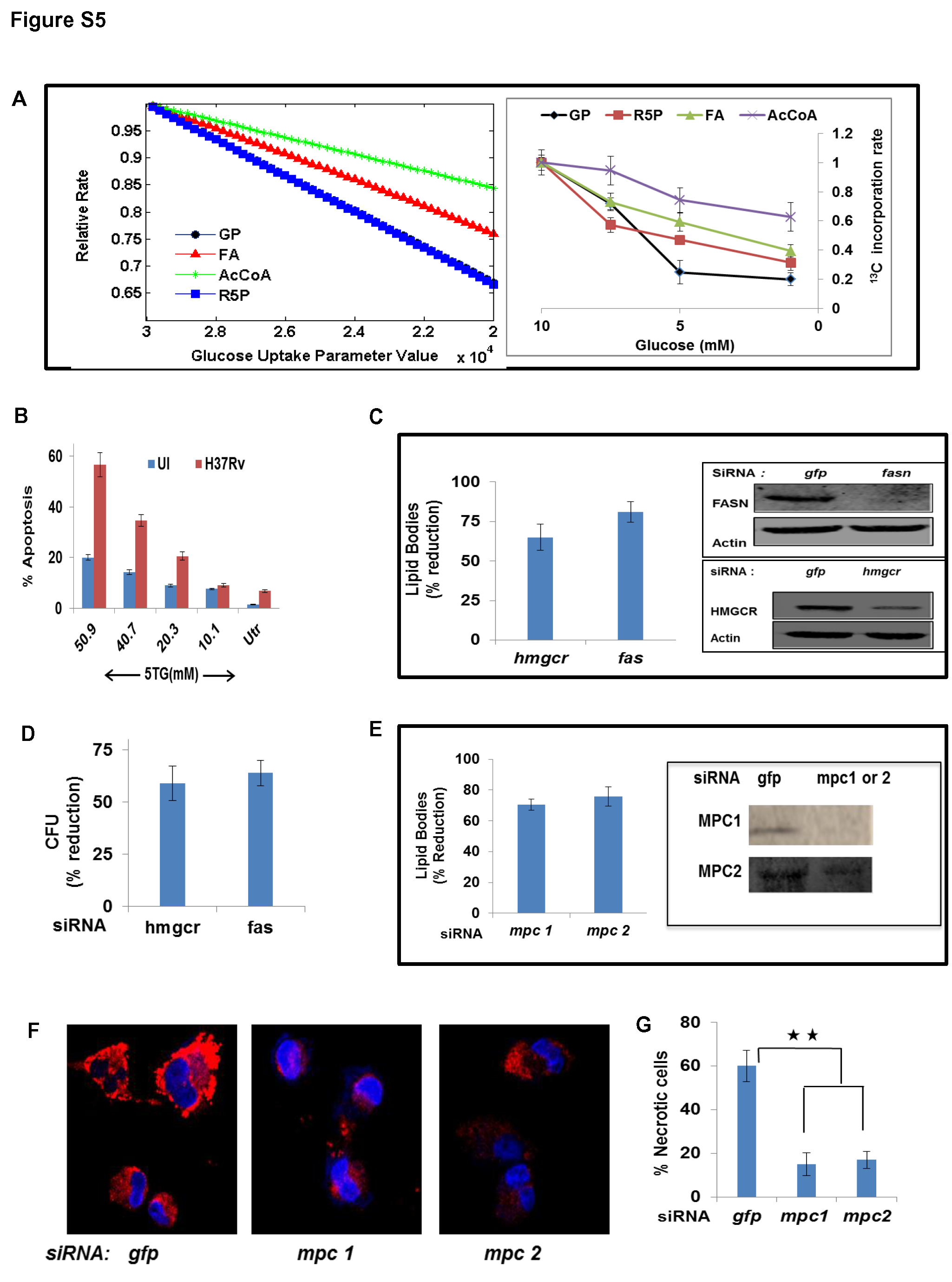

Supplement: Figure S5 — Model validation, lipid body accumulation, effect on bacterial CFU, and necrosis. A. The panel on the left depicts the results of a simulation exercise that probed for the effects of varying glucose uptake rates on GP, FA, AcCoA and R5P synthesis. Simulation was done on the ODE model derived for JAL2287-infected cells, using the parameter values for the 24 hr infection time point. The panel on the right gives the corresponding, experimentally derived, results wherein JAL2287-infected cells (24 hr p-i) were treated with decreasing concentrations of glucose in the culture medium. Values are mean ±SE obtained from at least 3 independent experiments. A good correspondence in trends between the two sets of results is evident. B. Effect of glycolysis inhibition on apoptosis in infected and UI primary human monocytes. UI and H37Rv infected cells were either left untreated or were treated with increasing concentrations of 5TG. Cells were scored for percentage of apoptotic cells at 24 hours p-i. (n = 3, mean ±SD). C–D. RNAi-mediated silencing of HMGCR or FAS inhibits LB accumulation and CFU. (C) Results are shown as percent reduction in LB accumulation in H37Rv-infected cells, relative to that obtained in cells mock-transfected with GFP-specific siRNA. Data are from one of three independent experiments, and represent an average of 200 cells ±S.E. (D) The corresponding effect on bacterial CFU values, in terms of percent reduction from that in mock siRNA-transfected (GFP-specific) cells. The efficiency of silencing was determined by Western blotting for both the proteins after silencing (FASN; HMGCR). E–G. RNAi-mediated silencing of either MPC1 or MPC2 on E) LB accumulation and G) necrosis in H37Rv- infected cells. Data represent an average of 200 cells and are presented in terms of percent reduction relative to the corresponding value in GFP-silenced cells; n = 3, mean ±SD , **p<0.01). F) Representative confocal images obtained after Lipid Tox staining are also shown. The eff [file ppat.1004265.s005.tif]

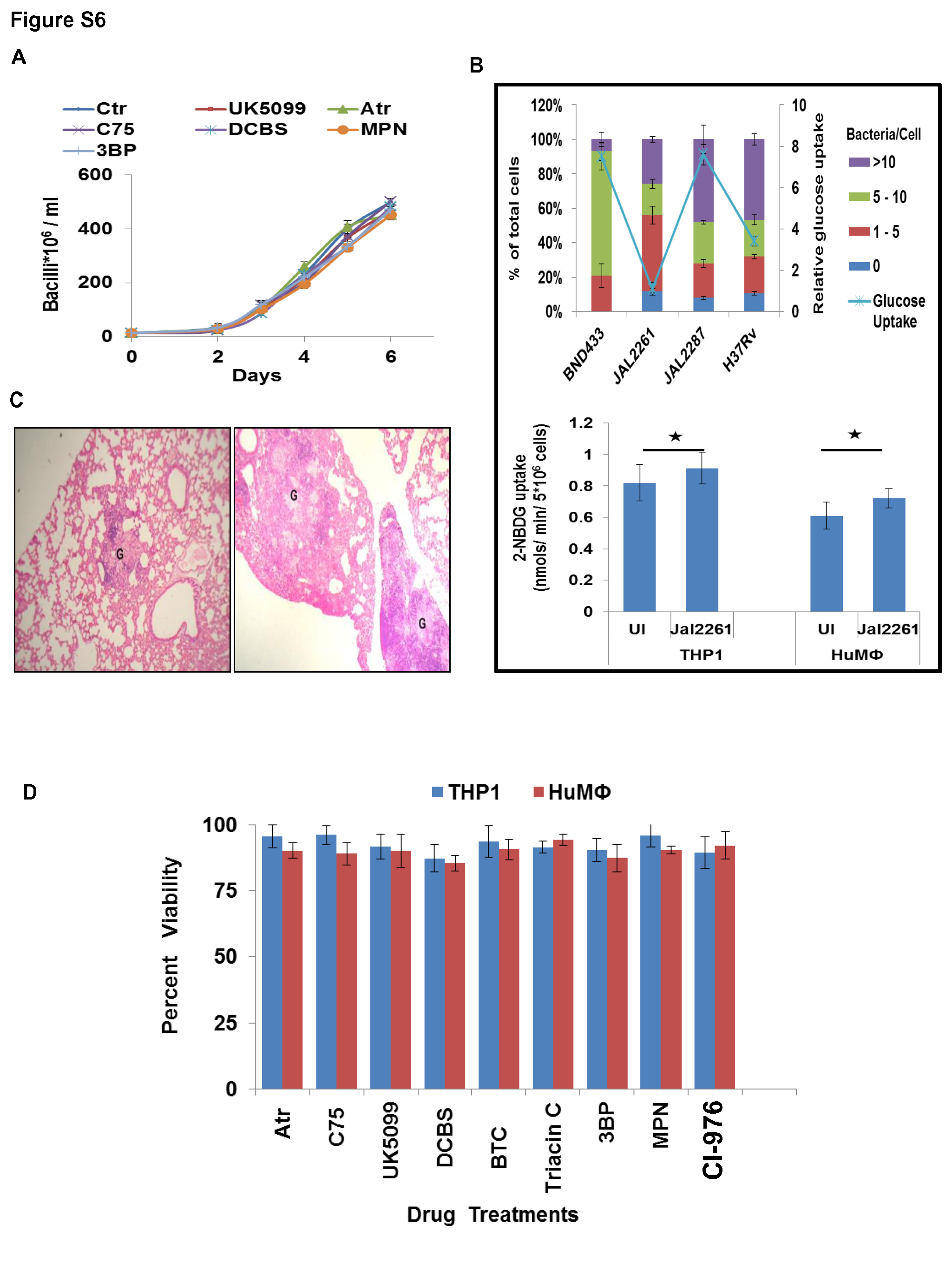

Supplement: Figure S6 — Inhibitor treatment of cells and free bacterial cultures. Glucose uptake efficiency and bacterial load. A. H37Rv was grown in liquid culture (7H9 medium) either in the absence or presence of the indicated drugs. At the indicated time points the bacterial growth was determined in terms of the O.D. values. (n = 3, mean ±S.D.). Inhibitors were used at the following concentrations: UK5099-5 µM; Atr-10 µM; C75- 20 µM; DCBS-50 µM; BTC-200 µM; MPN-100 nM, 3BP at 50 µM (n = 3 mean ±SE). B. Glucose uptake efficiency. The upper panel depicts the bacillary load per cell for individual strains obtained by 6 hours of infection. In the graph, the bars represent the percentage of total cell population harboring the indicated range of bacillary load (from 0 to >10 bacilli per cell). The Z axis represents the alteration in glucose uptake inflicted by the virulent strains compared to UI cells at 24 hr p-i. Lower panel: Infection with JAL2261 does not induce an increase in glucose uptake in either THP-1 or HuMФ cells. The rate of 2-NBDG uptake was monitored at 24 hours p-i. (n = 3 ,mean ±SD, *p>>0.05). C. Photomicrograph showing representative hematoxylin and eosin-stained lung sections of mice infected with H37Rv containing a small well defined granuloma (G) (left panel); and JAL2287 displaying two large granulomas (G) (right panel). D. Uninfected THP-1 and HuMФ cells were treated individually with UK5099 5 µM; Atr-10 µM; C75- 20 µM; DCBS-50 µM; BTC-200 µM; MPN at 100 nM, CI 976-1 µM; Triacin C at 5 µM; 3BP- 50 µM for 48 hrs and the percent viability over untreated cells was determined using the MTT assay (n = 5, mean ±S.D.). (TIF) [file ppat.1004265.s006.tif]

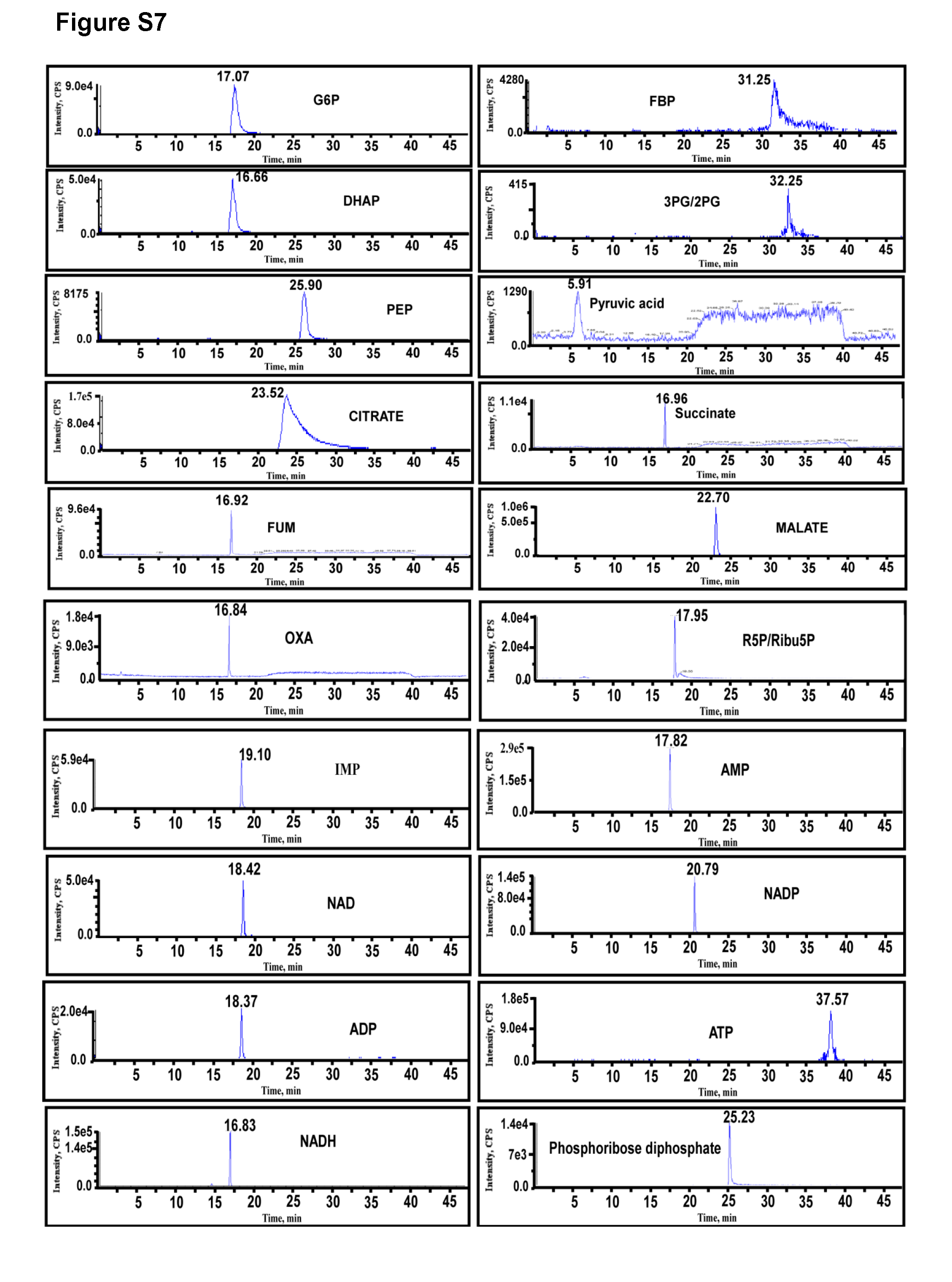

Supplement: Figure S7 — Extracted ion chromatography (XIC) of standards. Chromatograms show the XIC of G6P, FBP, DHAP, 3PG/2PG, PEP, Pyruvic acid, CITRATE, Succinate, FUM, MALATE, OXA, R5P/Ribu5P, IMP, AMP, NAD, NADP, ADP, ATP, NADH and PRD as purified standards (1 µM) and their retention time as obtained after resolution on an Agilent Polaris 5 NH2 2×150 mm column. (TIF) [file ppat.1004265.s007.tif]

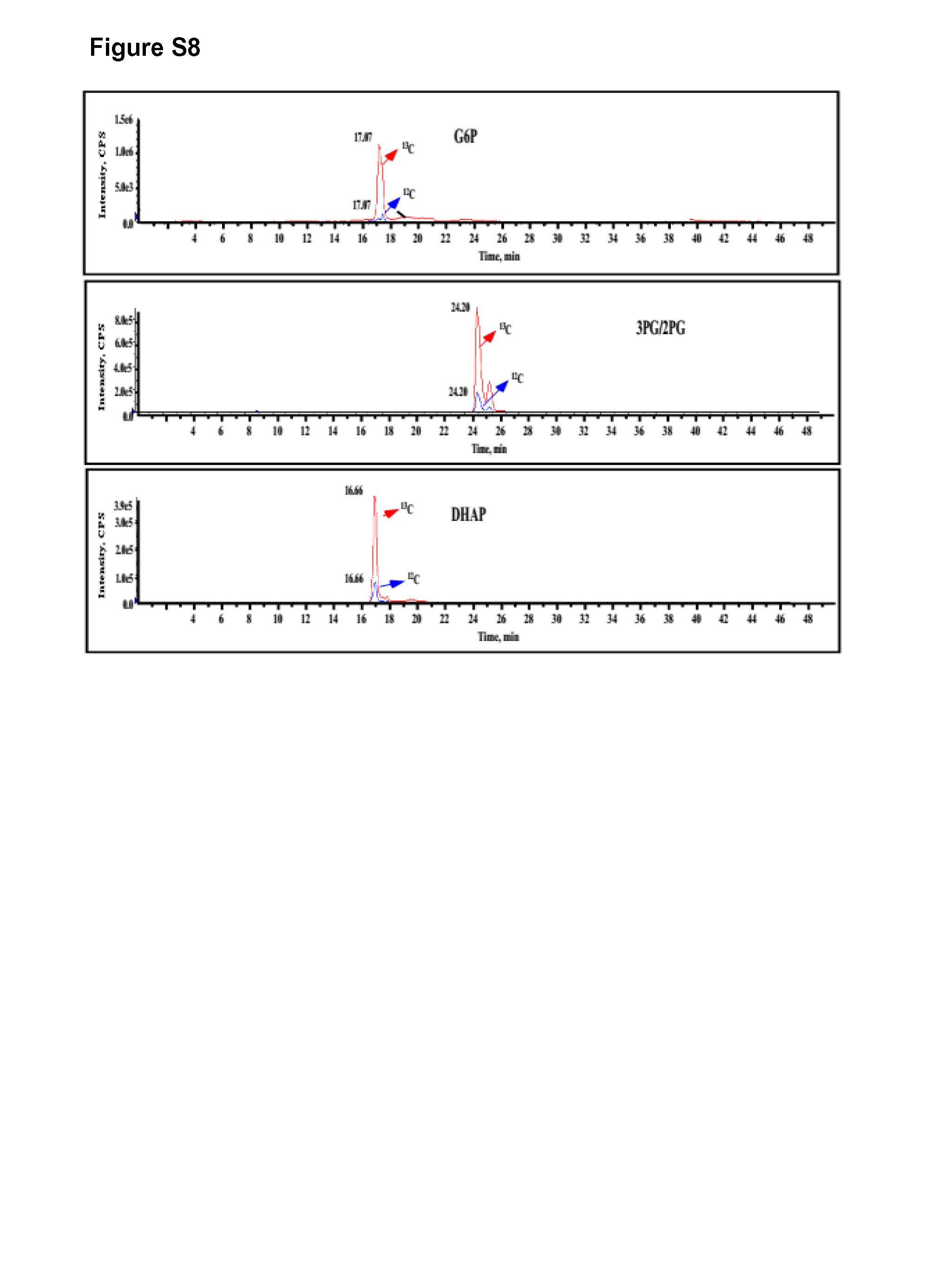

Supplement: Figure S8 — XIC of G6P; 3PG/2PG; DHAP. Figure depicting XIC (extracted ion chromatography) of G6P, transition 259→97, represent 12C and 265→97, represent 13C G6P; m/z 185→79, represent 12C and 188→79, represent 13C 3PG/2PG and m/z 169→97, represent 12C and 172→97, represent 13C DHAP. (TIF) [file ppat.1004265.s008.tif]

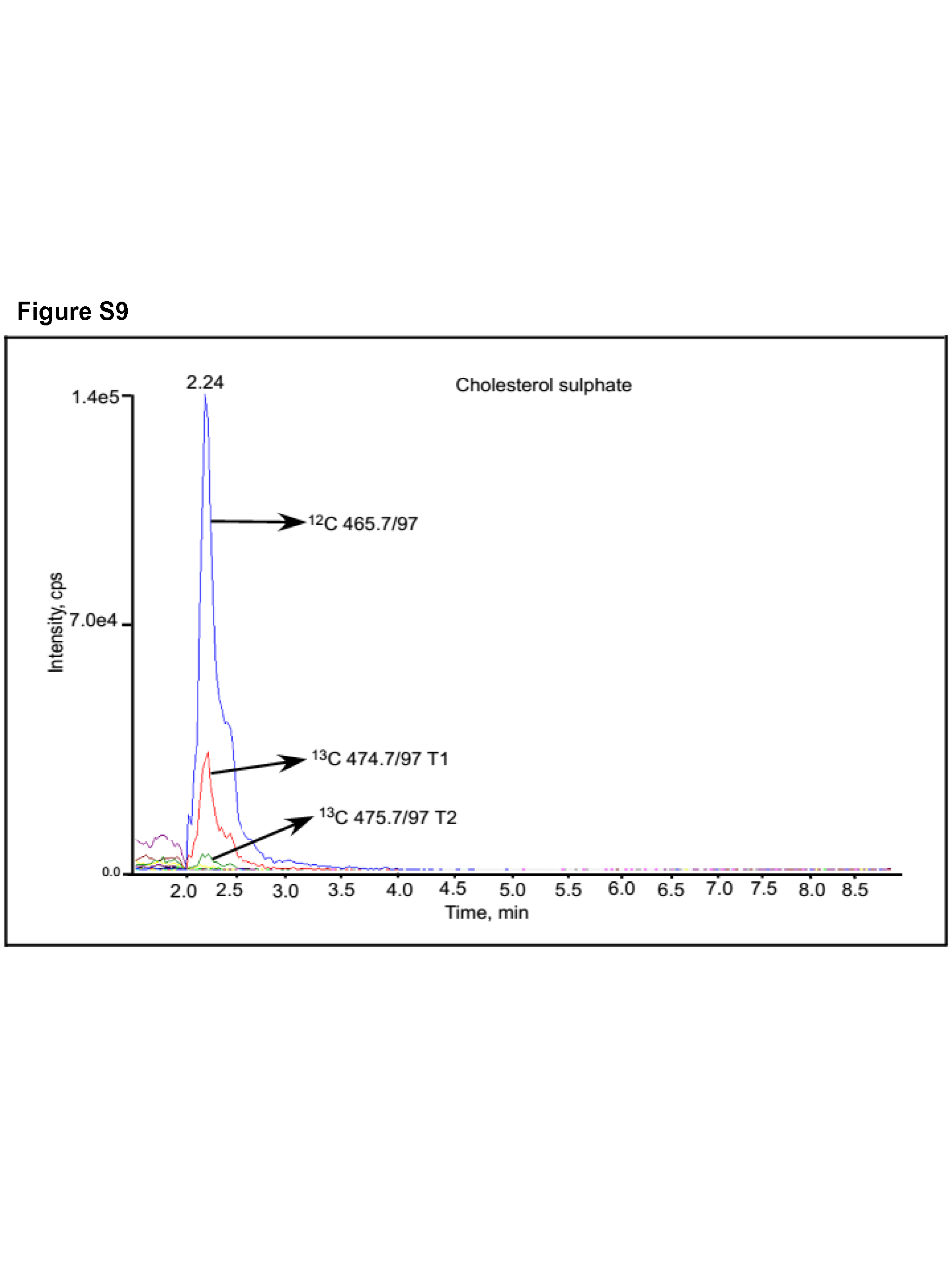

Supplement: Figure S9 — LC-MS/MS profile of cholesterol sulfate. Representative LC-MS/MS results of co-eluted 12C and 13C carbon spectra for cholesterol sulfate (synthesized), which were identified by Luna CN column (100 Å, 2×150 mm 3 µ, Phenomenex, Torrance, CA, USA). 12C species is shown by m/z 465.7→97 and 13C by m/z 474.7→97 and 475.7→97. (TIF) [file ppat.1004265.s009.tif]

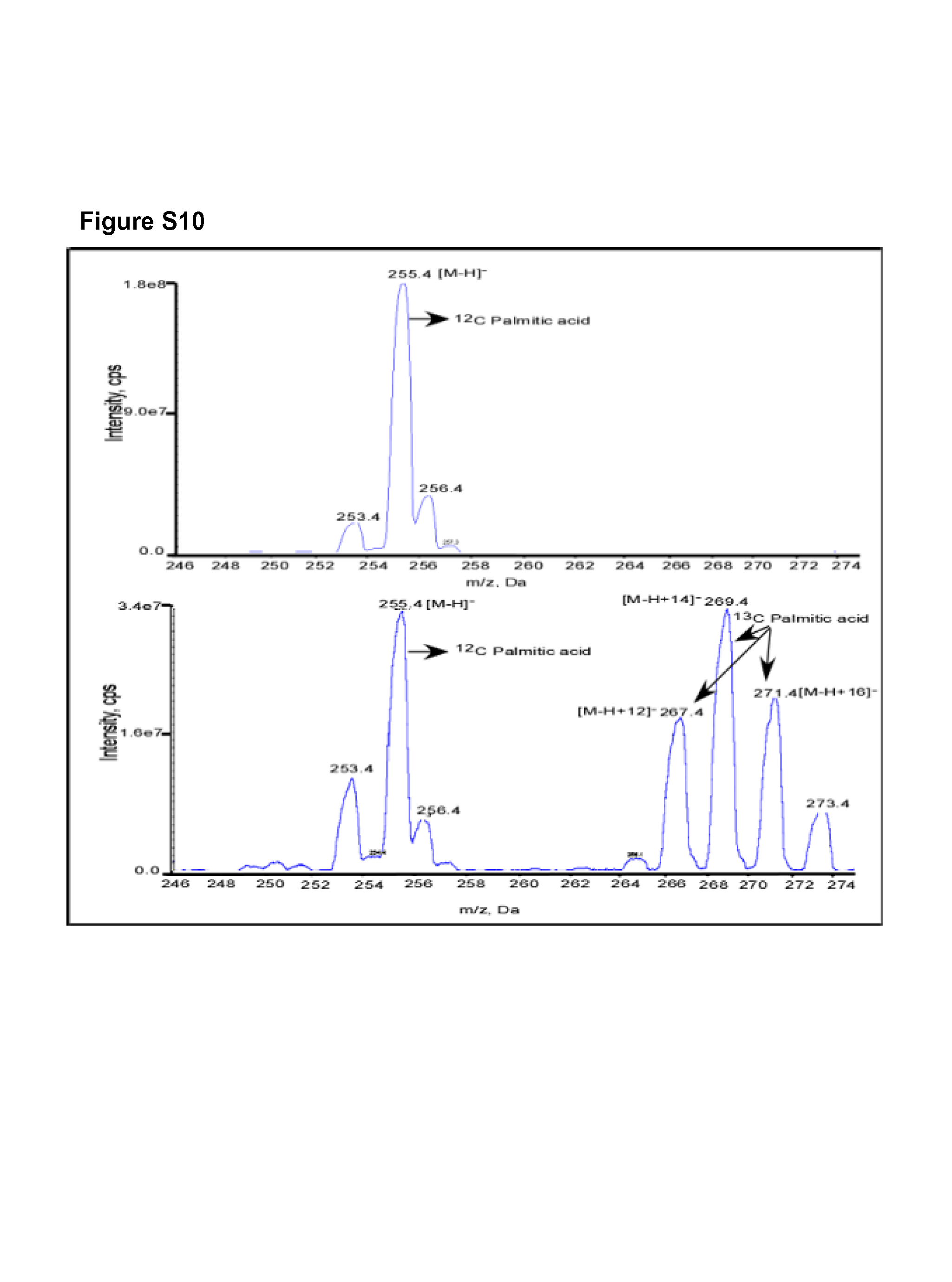

Supplement: Figure S10 — Ion spectra of free fatty acid obtained by direct infusion. Representation of free fatty acids by the direct infusion method. A) standard palmitic acid spectra observed at m/z 255.4 in negative polarity. B) Representation of sample (Free fatty acids) spectra, 12C palmitic acid observed at m/z 255.4 and its respective 13C at m/z 267.4, 269.4 and 271.4 were convincingly identified and quantitated. (TIF) [file ppat.1004265.s010.tif]
