# Supplementary material for: Pathogenicity of Mycobacterium tuberculosis Is Expressed by Regulating Metabolic Thresholds of the Host Macrophage
Source: PLoS Pathog. 2014 Jul 24;10(7):e1004265. doi: 10.1371/journal.ppat.1004265 (PMC4110042; doi:10.1371/journal.ppat.1004265)
Supplement: Methods S1 — The document provides detailed information on the materials used, the experimental protocols followed, the methodology optimized for LCMS/MS analysis of metabolite samples, and the mathematical model employed with its validations. (DOCX) [file ppat.1004265.s011.docx]

**SUPPLEMENTARY METHODS –S1**

**Materials:**

Glucose free RPMI 1640, Glutamine free RPMI 1640, JC1 Mitochondrial staining Kit, Atorvastatin Calcium, ^13^C_5_ Glutamine (99%), 1,2,3 benzene tricarboxylate, Phloretin, UK5099, 2-Deoxy-D-Glucose , 5-Thio-D-Glucose , Glucose 6-phosphate , Fructose 6-phosphate , Fructose bisphosphate , Glyceraldehyde 3-phosphate, Dihydroxyacetone phosphate, 3-Phosphoglycerate, 2-Phosphoglycerate, Phosphoenolpyruvate , Pyruvate , Lactate, Citrate, Aconitate, α-Ketoglutarate , Succinate, Fumarate, Malate, Oxaloacetate , Ribulose 5-phosphate , Phosphoribose diphosphate , Inosine monophosphate, Adenosine monophosphate, Nicotinamide adenine dinucleotide phosphate, Mevalonate, Guanosine monophosphate, AcetylCoA, MalonylCoA , HMGCoA, Butyric acid, Adenosine diphosphate , Adenosine triphosphate , Nicotinamide adenine dinucleotide, Nicotinamide adenine dinucleotide phosphate, Glutamate, Orotic acid, Dihydroorotic acid, Tryptophan, Cholesterol, Palmitic acid, Pyridine sulfurtrioxide complex, Pyridine , Ethyl acetate and Hexane were purchased from Sigma. ^13^C_6_ Glucose(99%) was bought from Eurisotop. HCS lipidtox Red neutral Lipid stain, 2-NBDG, Rabbit pAB to GLUT3-FITC, Goat pAB to Ms IgG-FITC were purchased from Molecular Probes (Invitrogen). Mouse mAB to GLUT-1, Rabbit pAB to FASN, Rabbit pAB to HMGCR, Rabbit pAB to MTCO2, Triacylglyceride quantification kit were from Abcam. Dharmacon siGENOME siRNAs against MPC-1, MPC-2, HMGCR, FAS were procured from Thermo Scientific. C75, Rotenone were obtained from Tocris. 3-Bromopyruvate(3BP) was from Alfa-Aesar. 3,5-dicholoro-2-hydroxy-N-(4-methoxy-biphenyl-3-yl)bezene sulfonamide (DCBS) was from Revelations Biotech (India). Acetonitrile (HPLC grade), Formic acid (LC/MS grade), and water (LC/MS grade) were purchased from Fisher Scientific (Fisher Scientific).

**Primary human PBMC-derived macrophages**

Heparinized human blood diluted 1:1 with RPMI1640 was layered onto equal volume of Ficoll Paque (Himedia) followed by centrifugation at 1600 rpm for 30 min. The PBMC layer formed at the interface was collected carefully and washed twice with RPMI. The cells were diluted in RMPI medium (without serum) to a concentration 2 ×10^6^/ml and 10 ml of diluted cells were put into a 75-cm^2^ tissue culture flask, and incubated for 2 hrs in a humidified 37°C incubator. The non-adherent cells were removed by aspiration, followed by two washes with RPMI. Complete media (with 10% FCS) was added and the cells were allowed to spontaneously differentiate into macrophages for 4 days in a humidified 37°C, 5% CO_2_incubator.

**MTT Assay**

Cells were washed with phenol red-free RPMI and 100 μl of the same was added to each well. 20 μl of MTT (5 mg/ml) was added and the cells were incubated for 45min at 37^o^ C followed by solubilization in DMSO (100 μl). Absorbance was recorded at a wavelength of 560nm.

**siRNA treatment**

Cells were treated with siRNA at a final concentration of 50nM using Dharmafect-2 transfection reagent (Dharmacon, ThermoFischer Scientific) according to manufacturer’s protocol. Media was changed once at 48 hours after siRNA treatment.

**Lipid and Cholesterol labeling Analysis**

Sample Generation: 6hours p-i or 36 hours p-i, 30*10^6^ cells were switched to labeled ^13^C_6_-glucose media for 4 hours. Parallel sets of unlabeled controls were also maintained. On completion of labeling duration, labeled and unlabeled cells were lysed with a mild lysis buffer – 0.1M Potassium Phosphate, 0.05 NaCl, 5mM Cholic Acid, 0.1% triton X-100 and the lysate was collected. The collected lysate was then vortexed and Centrifuged at 3000g, 10 minutes, 4°C .

Lipid Extraction and Cholesterol derivatization: Lipid extraction was performed as per the protocol of Bligh and Dyer[[1](#_ENREF_1)]. The extract was dried under a stream of nitrogen gas and stored in dark at -20°C until further use for Lipid Hydrolysis and cholesterol sulfation. Hydrolysis was performed on the extracted lipids to release free fatty acids .Cholesterol was converted to cholesterol-3-sulphate for better ionization on MS.

Acid Hydrolysis: Hydrolysis was performed as previously mentioned[[2](#_ENREF_2)] with slight modifications. The dried lipid extracts were re-suspended in 100ul H_2_O and 1ml of 4:1 Acetonitrile: 37%(v/v) Hydrochloric acid was added followed by incubation at 90°C for 2 hours. The extracts were cooled to room temperature and 1ml of Hexane was added followed by vortexing . Samples were left at RT for 5 minutes followed by centrifugation at 3000g and The supernatant was the hydrolyzed lipid layer. The pellet obtained was extarcted with Hexane twice and the supernatants were pooled. The extract was divided into two equal halves. One half was dried under a stream of nitrogen, re-suspended in 200µl of 50:40:5 Chloroform: methanol: water and 0.01% aqueous Ammonia and used directly for Lipid analysis.

Cholesterol Derivatization: The second half was used for cholesterol derivatization and estimation. In a fresh glass vial, 2.5mg of Sulfur trioxide pyridine was added followed by 0.5mg of dry Pyridine. Sonication was done for 10 seconds in a water bath followed by the addition of 20µl of the sample to the vial and sonication for 10 seconds followed by incubation at RT for 15 minutes. 2.1µl of 314mM solution of Barium Acetate was added to the vial and sonication was performed for 10 seconds followed by incubation at RT at 10minutes.Samples were then incubated at 4°C for 60 minutes. After the incubation, 120µl of Methanol was added and mix was centrifuged at 13000g for 10 minutes. The supernatants thus obtained were used directly for Mass spectrometric measurement of Cholesterol.

**Metabolite determination in the mitochondrial and cytoplasmic fractions.**

Digitonin based lysis method was used to isolate intact mitochondrion from cells[[3](#_ENREF_3)] with slight modifications. Briefly, 5*10^6^ Cells were labeled kinetically with ^13^C_6_-glucose for 0,2,5,15,30 and 60 minutes. After labeling, media was removed from the culture dish and 100µl of lysis buffer (0.25M Sucrose, 10mM pH 7.0 MOPS, 3mM EDTA and 0.2mg/ml Digitonin) was added to each well for 1 minute, the lysate was then collected and mitochondria were separated by centrifugation at 3000g at 4°C for 10 minutes. Supernatant was stored as the cytoplasmic extract and the pellet containing the mitochondria was washed with PBS. The supernatant obtained was pooled with the cytoplasmic extract while the pellet contained the mitochondrial fraction. The Extracts were dried, re-suspended in MS Grade water and analyzed by LC-MS/MS. The purity of the mitochondrial and cytoplasmic extracts was determined by Western blotting.

**Isolation of bacterial metabolites.**

Bacilli were isolated from 30*10^6^ infected cells (MOI 10:1) at 12, 24, 48 hours p-i. For this cells were labeled for 2, 5, 15 30, 60 minutes with ^13^C_6_-glucose. On completion of incubation, the cells were lysed with 0.1% Triton X-100. The lysate was centrifuged at 300 g for 10 min at 4°C to remove host cell debris. The supernatant containing the bacteria was re-centrifuged at 4000g for 10 minutes at 4°C to pellet the bacteria[[4](#_ENREF_4)] . The supernatant so obtained was used to probe host labeling. The pellet was re-suspended in ice cold PBS and bead beating was performed. The lysate so obtained was centrifuged at 300g, 5 minutes at 4°C to pellet the debris. The supernatant was dried and was analyzed for bacterial metabolite labeling patterns.

**Staining and Confocal Microscopy**

JC1 Staining: JC1 Mitochondrial Staining Kit for mitochondrial potential changes detection was obtained from Sigma. For staining, cells were overlaid with staining solution and incubated at 37°C, 5% CO2 for 30 minutes. At completion of incubation, the staining solution was removed; cells were washed twice with growth media and overlaid with complete growth media. Stained cells were observed under confocal microscope.

*Lipid Tox staining*: Lipid droplets were imaged and quantified using LipidTOX™ neutral lipid stains LipidTOX™ neutral lipid stain (Molecular Probes). The staining was performed according to manufacturer’s protocol for fixed cells.

*GLUT1/GLUT3 Staining*: Fixed cells were permeabilized with 0.2%(v/v) Triton X-100 for 20 min, washed with PBS and blocked with 3% (w/v) BSA( in PBS) for 60 min. The cells were washed thrice with PBS and GLUT-1 Mouse Ab (AbCam) or GLUT3-FITC Rabbit Ab, at a 1:100 dilution, was added for 60 min at room temperature. Cells were washed with PBS (thrice). Alexa Fluor488 goat anti mouse (Invitrogen), at 1:200 dilution, was added to wells with GLUT-1 Antibody for 45 min at room temperature. Cells were washed with PBS (thrice). The coverslips were mounted on slides with Slow fade (Invitrogen).

*DAPI staining*: The cell nuclei were stained using 300nM DAPI solution (in H2O) for 5 min and then washed.

**Mass Spectrometry (MS)**

**MS parameters**

As described in Methods, MS parameters for metabolites under study can be found in Table-S2. In the negative polarity mode, [M-H]^-^ was the dominant parent ion for almost all of the compounds investigated due to the loss of a proton. This was followed by decarboxylation as the preferred fragmentation for most of the organic acids except for malate. Malate was found to first lose water molecule followed by decarboxylation to produce m/z= 71[M-H-18-44]^-^. Fumarate, an isomer of malate, tends to produce the same species in MRM spectrum producing noticeable interference with the malate peak, observed at 22.70 min. For metabolites containing phosphate moieties the main product ion was [M-H]^-^ (parent) and either 97 [H_2_PO_4_]^-^ or 79 [PO_3_]^-^. This LC-MS/MS method could also resolve the important redox cofactors such as NAD and NADP by monitoring the loss of nicotinamide moiety, NADH through loss of [PO_3_]^-^. Energy molecules such as ATP, ADP, AMP were monitored by the loss of [PO_3_]^-^.

**Quantification and validation of metabolite data.** Primary stock solution mix containing each of the metabolite at a final concentration of 1mM was prepared in 15% ACN. The working solution was prepared by diluting the stock to a concentration of 100µM and this was used for calibration of standards and quality control (QC). The calibration curve was obtained by injecting the standard solution individually at five different selected concentrations, ranging from 500nM to 10µM (500nM, 1µM, 2µM, 5µM, 10µM, data not shown). Calibration curves were constructed by plotting the area under the peak against the concentration of the compound, weighted linear regression was used to fit the calibration curve. As shown in Table S3, the calibration curves showed very good linearity with high correlation coefficients (R^2^) close to 1. A representative panel for 20 metabolites is depicted in Figure S7. The peak areas for different metabolites obtained on LC-MS were converted to concentration by normalizing against the peak areas of respective MS standards. (Table S3). To normalize for extraction efficiency, samples were spiked with Fumaric acid- ^13^C_4_d_4_ before extraction.

The validated calibration curves were used successfully in the routine analysis for the characterization of the intracellular metabolite concentration in cell extracts. Further, areas under the peaks for individual metabolites were determined and the concentration of each of these metabolites was calculated using the standard curves generated. The retention time, concentration ranges of standards used and R^2^  are depicted Table-S3.

**Monitoring the CCM pathways by ^13^C_6_-Glucose labeling.**

To further interrogate central carbon metabolism, the time dependent conversion of [U-^13^C] glucose, in THP-1 cells, to other metabolites was monitored using targeted liquid chromatography mass spectrometry (LC/MS/MS). MRM profiles for individual metabolites were generated by monitoring ^12^C and their respective ^13^C transitions. The transitions used for the metabolites under study are mentioned in Figure S2B. Most of the metabolites showed excellent resolution of the ^13^C labeled metabolite from their co-eluting ^12^C-labeled counterpart. Representative spectra for G6P, 3PG/2PG and DHAP are shown in Figure S8

For LC-MS/MS, the Agilent Zorbax Eclipse Plus C18, narrow bore (2.1 × 150 mm 5µ) LC column was used for the quantitation of AcCoA and MaCoA. The reverse-phase HPLC separation of metabolites was achieved using a linear gradient from 0-60% B over 20min where solvent A was 10mM tributylamine in H_2_O adjusted to pH 4.95 with 15mM acetic acid and solvent B was neat methanol. AcetylCoA and MalonylCoA showed excellent retention in the C18 column and eluted at the end of the gradient (60% ACN).

Luna CN column (100Å, 2 x150 mm 3µ ,Phenomenex, Torrance, CA, USA) was used for cholesterol sulphate separation and MS/MS quantitation. Reverse-phase HPLC was performed using a linear gradient 80-99%B over 20 min and the gradient held at 99%B for another 20mins. Solvent A was 97:3 water:methanol containing 10mM tributylamine, 15mM acetic acid final pH 4.95. Solvent B was 100% methanol. SRM was optimized 465.7/ 97 as parent and product ions.

For quantification and validation 0.05µM to 2 µM (0.05, 0.1, 0.5, 1, and 2 µM) of cholesterol sulphate standard was injected and a calibration curve was constructed. The calibration curve showed a good linearity with a high correlation coefficient of 0.9. To capture ^13^C label incorporation into Cholesterol, MRM was generated by taking appropriate ^13^C transition of cholesterol sulphate, detected in mass spectrometry (table S4). A representative mass spectrum for cholesterol sulphate with different ^12^C and ^13^C transitions is shown in Figure S9. The various unlabeled and labeled, co-eluting cholesterol moieties were distinguished in the mass spectrometry by distinct mass. The MRM profile was generated in negative polarity since cholesterol sulphate readily provides sulphate ions.

**Analysis of FA by the direct infusion method.**

Fatty acid analysis was performed using direct infusion method in negative polarity. Scan type Q1 were selected to monitor fatty acids present in sample. For standardization palmitic acid [m/z=255.42 (M-H)^-^] standard at 10µM concentration was used. The total run time of 100 cycles were optimized with standards. A representative spectrum of palmitic acid standard is shown in the Figure S10 (upper panel). The optimized source parameters selected were ionisation spray voltage (IS) -2400V, CUR, GS1, GS2, 10, 10, 3, respectively (Volts). The compound dependent parameters obtained were DP, EP -80V, -10V respectively. Mass range of 100-400 Dalton (Da) with step size 0.1 Da were selected. Samples were injected in to the Mass Spectrometer with the help of model 11 PLUS syringe pump (Harvard apparatus, Holliston, USA) at the flow rate of 10µl/min. Spectra showed clear distinction between ^12^C and  ^13^C- labeled palmitate. A representative run is shown Figure S10

**Mathematical Modelling**

**Mathematical model:**

*d[x_gi_] /dt = K-r_0_ [x_gi_],*

*d[x_G6P_] /dt = k_1_-δ_1_ [x_G6P_] +r_0_ [x_gi_]-r_1_ [x_G6P_]-r_2_ [x_G6P_],*

*d[x_FBP_] /dt = k_2_-δ_2_ [x_FBP_] +r_1_ [x_G6P_]-r_3_ [x_FBP_],*

*d[x_DHAP_] /dt = k_3_-δ_3_ [x_DHAP_] +2r_3_ [x_FBP_]-r_4_ [x_DHAP_]+δ_14_ [x_R5P_],*

*d[x_PG_] /dt = k_4_-δ_4_ [x_PG_] +2r_4_ [x_DHAP_]-r_5_ [x_PG_],*

*d[x_PEP_] /dt = k_5_-δ_5_ [x_PEP_]-2r_6_ [x_PEP_]+r_5_ [x_PG_],*

*d[x_Pyruvate_] /dt = k_6_-δ_6_ [x_Pyruvate_] +2r_6_ [x_PEP_]-r_7_ [x_Pyruvate_],*

*d[x_Acoa_] /dt = k_7_-δ_7_ [x_Acoa_]+r_7_ [x_Pyruvate_]-(r_8+_r_18_+r_19_+r_20_) [x_Acoa_]+r_9_ [x_citrate_],*

*d[x_citrate_] /dt = k_8_-δ_8_ [x_citrate_]+ r_8_ [x_Acoa_][x_oxa_]-(r_9_+r_10_) [x_citrate_],*

*d[x_AKG_] /dt = k_9_-δ_9_ [x_AKG_]- r_11_ [x_AKG_]+r_10_ [x_citrate_],*

*d[x_Succinate_] /dt = k_10_-δ_10_ [x_Succinate_]+ r_11_ [x_AKG_]-r_12_ [x_succinate_],*

*d[x_Fumarate_] /dt = k_11_-δ_11_ [x_Fumarate_]+ r_12_ [x_succinate_]-r_13_ [x_Fumarate_],*

*d[x_Malate_] /dt = k_12_-δ_12_ [x_Malate_]+ r_13_ [x_Fumarate_]-r_14_ [x_Malate_],*

*d[x_OXA_] /dt = k_13_-δ_13_ [x_OXA_]+ r_14_ [x_Malate_]-r_8_ [x_OXA_]+r_9_ [x_citrate_],*

*d[x_R5P_] /dt = k_14_-δ_14_ [x_R5P_]+ r_2_ [x_G6P_]-r_15_ [x_R5P_],*

*d[x_IMP_] /dt = k_15_-δ_15_ [x_IMP_]+ r_15_ [x_R5P_]-(r_16_+r_17_) [x_IMP_],*

*d[x_AMP_] /dt = k_16_-δ_16_ [x_AMP_]+ r_16_ [x_IMP_],*

*d[x_GMP_] /dt = k_17_-δ_17_ [x_GMP_]+ r_17_ [x_IMP_],*

*d[x_MVA_] /dt = k_18_-δ_18_ [x_MVA_]+ r_18_ [x_Acoa_],*

*d[x_Malonylcoa_] /dt = k_19_**-δ_19_ [x_Malonylcoa_]+ r_19_ [x_Acoa_],*

*d[x_HB_] /dt = k_20_-δ_20_ [x_HB_]+ r_20_ [x_Acoa_],*

The above set of ODEs describes the model shown in Figure 2A.

**Predictions and validations**: For validation, the estimations were then crosschecked experimentally with the following parameters.

*A) Glucose uptake rate*

We estimated the glucose uptake rate from other parameters obtained experimentally. For this we hypothetically tested different values for glucose uptake rate and chose the one that fit best in predicting the saturation concentration. The predicted uptake rates for different strains at different time points with respect to uninfected cells are given in the table S6. The table reflects an increase in the Glucose uptake capacity of cells infected with the Virulent strains. To test the hypothesis, the predicted values were compared with the experimental glucose uptake rates (as depicted in Figure 3A). Here too the Uptake rate of Glucose increased in the cells infected with the virulent strains. Table S7 details the experimentally obtained values for glucose uptake

1. *Lipid Synthesis*

To further substantiate our proposed model we predicted the synthesis rate of cholesterol and fatty acid for different strains of bacteria at different time points. The predictions were made with the parameter representing the outward flux from MVA and Malonyl-Coa, which were obtained after the estimation (Table S8). The table shows that H37Ra and *M.smeg* are close to uninfected rate, while in the other three strains there is an increase in the rate of synthesis of fatty acid/ cholesterol with respect to uninfected. In JAL2287, there is an early jump in both fatty acid and cholesterol synthesis rate, which goes down with time. In case of BND433 there is a huge increase in early cholesterol synthesis rate, which goes down with time while the synthesis rate of fatty acid increases with time. Finally in case of H37Rv, both cholesterol and fatty acid synthesis increases with time with more increase in cholesterol synthesis rate than fatty acid synthesis. To validate the result we obtained the experimental cholesterol and Fatty acid synthesis rate (Table S9)

*C) Energy Molecules*

We estimated the net Glycolytic ATP synthesis rate utilizing the formula derived as follows:

The energy production and consumption during glycolysis (i.e. from glucose to Pyruvate) was predicted using the following information about the glycolytic pathway

1. One molecule of ATP is consumed From Glucose to G6P
2. from G6P to FBP: One molecule of ATP is consumed
3. From DHAP to PG: 2 molecules of NADH and two molecules of ATP are produced
4. From PEP to Pyruvate: 2 molecules of ATP are produced.

Thus , mathematically, using our model parameters, we have

Net ATP synthesis rate : -K-r1+2*r4+2*r6

**GP calculations:** Algorithm followed for the simulation exercise depicted in Figure 4B:

1. *X_i_* = synthesis rates of glycolytic metabolites for Uninfected cells

synthesis rates of glycolytic metabolites for virulent strain infected cells

(i represents the hours post infection at which metabolic flux was monitored)

1. Calculate mean and SD for all *X_i_’s_._*
2. *Y_i_* = synthesis rates of glycolytic metabolites for Modified Uninfected cells

synthesis rates of glycolytic metabolites for virulent strain infected cells

(Modification : altered glucose uptake rate and DHAP synthesis rate)

1. Calculate mean and SD for *Y_i_’s.*
2. Plot *X_i_’s_._*and *Y_i_’s* as time series graphs.

Similar simulations were carried out with H37Ra to obtain figure 4C.

References

1. Bligh EG, Dyer WJ (1959) A rapid method of total lipid extraction and purification. Canadian journal of biochemistry and physiology 37: 911-917.

2. Aveldano MI, Horrocks LA (1983) Quantitative release of fatty acids from lipids by a simple hydrolysis procedure. Journal of lipid research 24: 1101-1105.

3. Sánchez-Alcázar JA, Ruı́z-Cabello J, Hernández-Muñoz I, Pobre PS, de la Torre P, et al. (1997) Tumor necrosis factor-α increases ATP content in metabolically inhibited L929 cells preceding cell death. Journal of Biological Chemistry 272: 30167-30177.

4. Beste DJ, Noh K, Niedenfuhr S, Mendum TA, Hawkins ND, et al. (2013) (13)C-Flux Spectral Analysis of Host-Pathogen Metabolism Reveals a Mixed Diet for Intracellular Mycobacterium tuberculosis. Chem Biol 20: 1012-1021.
