# Supplementary material for: Pathogenicity of Mycobacterium tuberculosis Is Expressed by Regulating Metabolic Thresholds of the Host Macrophage
Source: PLoS Pathog. 2014 Jul 24;10(7):e1004265. doi: 10.1371/journal.ppat.1004265 (PMC4110042; doi:10.1371/journal.ppat.1004265)
Supplement: Table S1 — Gradient profile for LC-MS/MS method on Agilent Polaris 5NH2 2×150 mm column. (DOCX) [file ppat.1004265.s012.docx]

**Table S1: Gradient profile for LC-MS/MS method on Agilent Polaris 5NH_2_ 2x150mm column.**

| S NO | Time (min) | Eluent A (Vol%) | Eluent B (Vol%) |
| --- | --- | --- | --- |
| 0 | 0.00 | 15 | 85 |
| 1 | 5.00 | 35 | 65 |
| 2 | 7.00 | 35 | 65 |
| 3 | 12.00 | 55 | 45 |
| 4 | 14.00 | 55 | 45 |
| 5 | 19.00 | 75 | 25 |
| 6 | 21.00 | 75 | 25 |
| 7 | 26.00 | 100 | 0 |
| 8 | 36.00 | 100 | 0 |
