# Supplementary material for: Pathogenicity of Mycobacterium tuberculosis Is Expressed by Regulating Metabolic Thresholds of the Host Macrophage
Source: PLoS Pathog. 2014 Jul 24;10(7):e1004265. doi: 10.1371/journal.ppat.1004265 (PMC4110042; doi:10.1371/journal.ppat.1004265)
Supplement: Table S2 — Table represents metabolites, their fragment ion mass in negative polarity along with optimized compound dependent parameters used in MRM (multiple reaction monitoring). (DOCX) [file ppat.1004265.s013.docx]

**Table S2: Metabolites and their fragments obtained in negative polarity with optimized compound dependent parameters used for MRM (multiple reaction monitoring).**

| Compound | [M-H]^-^ | Main product ion | DP(V) | CE(V) | CXP(V) |
| --- | --- | --- | --- | --- | --- |
| G6P/F6P | 259 | 97 [H_2_PO_4_]^-^ | -50 | -25 | -6 |
| FBP | 339 | 97 [H_2_PO_4_]^-^ | -95 | -65 | -10 |
| DHAP/G3P | 169 | 97 [H_2_PO_4_]^-^ | -25 | -14 | -4 |
| 3PG/2PG | 185 | 79 [PO_3_]^-^ | -35 | -41 | -6 |
| PEP | 167 | 79 [PO_3_]^-^ | -40 | -40 | -9 |
| PYR | 87 | 43 -CO_2_ | -30 | -15 | -7 |
| CIT | 191 | 87 [C_2_H_3_OCOO]^-^ | -30 | -25 | -2 |
| SUC | 117 | 73 -CO_2_ | -20 | -30 | -5 |
| FUM | 115 | 71 -CO_2_ | -35 | -12 | -7 |
| MAL | 133 | 71 -CO_2_+H_2_O | -30 | -22 | -8 |
| OXA | 131 | 87 -CO_2_ | -25 | -12 | -10 |
| R5P/Ribu5P | 229 | 97 [H_2_PO_4_]^-^ | -33 | -28 | -7 |
| IMP | 347 | 97 [H_2_PO_4_]^-^ | -45 | -32 | -7 |
| AMP | 346.2 | 79 [PO_3_]^-^ | -70 | -62 | -7 |
| NAD | 662.3 | 540.1 Nicotinamide(C_6_H_6_N_2_O) | -50 | -22 | -9 |
| NADP | 742.2 | 620.1 Nicotinamide(C_6_H_6_N_2_O) | -60 | -22 | -12 |
| MVA | 147.2 | 103.2 -CO_2_ | -57 | -14 | -4 |
| GMP | 362.1 | 79 [PO_3_]^-^ | -60 | -43 | -20 |
| AcCoA | 808.6 | 79 [PO_3_]^-^ | -80 | -95 | -5 |
| MalonylCoA | 852.5 | 79 [PO_3_]^-^ | -25 | -100 | -5 |
| 3HB | 103 | 59 -CO_2_ | -60 | -25 | -8 |
| ADP | 426.1 | 79 [PO_3_]^-^ | -80 | -88 | -9 |
| ATP | 506.1 | 79 [PO_3_]^-^ | -90 | -106 | -10 |
| NADH | 664.2 | 79 [PO_3_]^-^ | -30 | -75 | -5 |
| PRD | 389 | 79 [PO_3_]^-^ | -45 | -72 | -9 |
