# Supplementary material for: Pathogenicity of Mycobacterium tuberculosis Is Expressed by Regulating Metabolic Thresholds of the Host Macrophage
Source: PLoS Pathog. 2014 Jul 24;10(7):e1004265. doi: 10.1371/journal.ppat.1004265 (PMC4110042; doi:10.1371/journal.ppat.1004265)
Supplement: Table S3 — Establishment of the LC-MS/MS method. Table lists the retention time and linearity of metabolite standard calibration curves (R2) over the concentration range of standards used. Calibration curves were generated by injecting each standard five times over a concentration range of 500 nM-10 µM. (DOCX) [file ppat.1004265.s014.docx]

**Table S3: Establishment of the LC-MS/MS method. Retention time and linearity of standards are shown in the table. Calibration curves were generated by injecting single standard five times in a concentration range of 500nM-10µM.**

| Compound | Retention time(min) | Linearity   \| Range \| R^2^ \| \| --- \| --- \| | |
| --- | --- | --- | --- | --- | --- |
| G6P | 17.07 | 1µM-10 µM | 0.97 |
| FBP | 31.25 | 1µM-10 µM | 0.96 |
| DHAP/G3P | 16.66 | 0.5 µM-5 µM | 0.96 |
| 3PG/2PG | 32.25 | 0.5 µM-10 µM | 0.91 |
| PEP | 25.90 | 0.5 µM-5 µM | 0.95 |
| PYR | 5.91 | 0.5 µM-10 µM | 0.93 |
| CIT | 23.52 | 1 µM-10 µM | 0.96 |
| SUC | 16.96 | 0.5 µM-10 µM | 0.93 |
| FUM | 16.92 | 0.5 µM-10 µM | 0.96 |
| MAL | 22.70 | 0.5 µM-10 µM | 0.95 |
| OXA | 16.84 | 1µM-10 µM | 0.98 |
| R5P/Ribu5P | 17.95 | 0.5 µM-10 µM | 0.92 |
| IMP | 19.10 | 0.5 µM-10 µM | 0.95 |
| AMP | 17.82 | 0.5 µM-10 µM | 0.93 |
| NAD | 18.42 | 1 µM-10 µM | 0.94 |
| NADP | 20.79 | 0.5 µM-10 µM | 0.92 |
| MVA | 12.40 | 1µM-10 µM | 0.98 |
| GMP | 22.68 | 1µM-10 µM | 0.98 |
| AcCoA | 24.22 | 0.5µM-10 µM | 0.96 |
| MalonylcoA | 22.13 | 0.5µM-10 µM | 0.98 |
| 3HB | 1.63 | 0.5µM-10 µM | 0.98 |
| ADP | 18.37 | 0.5 µM-10 µM | 0.94 |
| ATP | 37.57 | 1 µM-10 µM | 0.93 |
| NADH | 16.83 | 0.5 µM-10 µM | 0.92 |
| PRD | 25.23 | 1µM-10 µM | 0.93 |
