# Supplementary material for: Pathogenicity of Mycobacterium tuberculosis Is Expressed by Regulating Metabolic Thresholds of the Host Macrophage
Source: PLoS Pathog. 2014 Jul 24;10(7):e1004265. doi: 10.1371/journal.ppat.1004265 (PMC4110042; doi:10.1371/journal.ppat.1004265)
Supplement: Table S4 — Establishment of the LC-MS/MS method for cholesterol sulphate. 13C and 12C transition and regression coefficient for cholesterol sulfate (Cholsul) are shown. (DOCX) [file ppat.1004265.s015.docx]

**Table S4: Establishment of LC-MS/MS method for cholesterol sulphate.^13^C and ^12^C transition and regression coefficient for cholesterol sulphate (Cholsul) are shown.**

| Q1 mass | Q3 mass | Compound | Linearity | |
| --- | --- | --- | --- | --- |
|  |  |  | Range | R^2^ |
| 465.7 | 97 | Cholsul | 0.05µM-2 µM | 0.90 |
| 474.7 | 97 | Cholsul T1 |  |  |
| 475.7 | 97 | Cholsul T2 |  |  |
| 476.7 | 97 | Cholsul T3 |  |  |
| 477.7 | 97 | Cholsul T4 |  |  |
