# Supplementary material for: Pathogenicity of Mycobacterium tuberculosis Is Expressed by Regulating Metabolic Thresholds of the Host Macrophage
Source: PLoS Pathog. 2014 Jul 24;10(7):e1004265. doi: 10.1371/journal.ppat.1004265 (PMC4110042; doi:10.1371/journal.ppat.1004265)
Supplement: Table S5 — The table shows flux distribution analysis from experimental 13C incorporation and 12C consumption rates for uninfected cells. (DOCX) [file ppat.1004265.s016.docx]

**Table S5: Flux distribution analysis from experimental c13 and c12 slopes for uninfected cells.**
