# Supplementary material for: Pathogenicity of Mycobacterium tuberculosis Is Expressed by Regulating Metabolic Thresholds of the Host Macrophage
Source: PLoS Pathog. 2014 Jul 24;10(7):e1004265. doi: 10.1371/journal.ppat.1004265 (PMC4110042; doi:10.1371/journal.ppat.1004265)
Supplement: Table S6 — Table depicts the predicted glucose uptake rates as ratio over the corresponding rates obtained for uninfected cells. (DOCX) [file ppat.1004265.s017.docx]

**Table S6: Predicted glucose uptake rates as ratios with uninfected cells**

|  | 6 hours p-i | 12 hours p-i | 24 hours p-i | 36 hours p-i | 48 hours p-i |
| --- | --- | --- | --- | --- | --- |
| M.smeg/UI | 1.6954 | 2.1958 | 1.9731 | 1.3302 | 2.1184 |
| H37Ra/UI | 0.2483 | 2.8812 | 2.2201 | 2.2119 | 2.3946 |
| H37Rv/UI | 1.0383 | 2.6703 | 5.9938 | 7.5984 | 6.6709 |
| JAL2287/UI | 1.3308 | 5.4434 | 6.6709 | 8.4731 | 8.1649 |
| BND433/UI | 3.6123 | 3.3812 | 4.4438 | 7.0281 | 7.2921 |
