# Supplementary material for: Pathogenicity of Mycobacterium tuberculosis Is Expressed by Regulating Metabolic Thresholds of the Host Macrophage
Source: PLoS Pathog. 2014 Jul 24;10(7):e1004265. doi: 10.1371/journal.ppat.1004265 (PMC4110042; doi:10.1371/journal.ppat.1004265)
Supplement: Table S7 — Table showing experimentally determined glucose uptake rate as ratio with uninfected cells. (DOCX) [file ppat.1004265.s018.docx]

**Table S7: Experimentally obtained glucose uptake rate as ratio with UI**

|  | 6 hours p-i | 12 hours p-i | 24 hours p-i | 36 hours p-i | 48 hours p-i |
| --- | --- | --- | --- | --- | --- |
| M.smeg/UI | 1.973±0.02 | 1.443±0.12 | 1.612±0.11 | 1.433±0.05 | 1.744±0.121 |
| H37Ra/UI | 0.6108±0.051 | 1.626±0.151 | 1.538±0.178 | 1.411±0.092 | 1.611±0.82 |
| H37Rv/UI | 0.8137±0.491 | 3.218±0.132 | 3.434±0.1182 | 5.552±0.57 | 5.702±0.302 |
| JAL2287/UI | 2.0173±0.118 | 7.4077±0.129 | 7.644±0.49 | 8.494±0.413 | 7.1±0.494 |
| BND433/UI | 2.501±0.15 | 2.614±0.442 | 7.512±0.65 | 7.012±0.651 | 6.882±0.442 |
