# Supplementary material for: Pathogenicity of Mycobacterium tuberculosis Is Expressed by Regulating Metabolic Thresholds of the Host Macrophage
Source: PLoS Pathog. 2014 Jul 24;10(7):e1004265. doi: 10.1371/journal.ppat.1004265 (PMC4110042; doi:10.1371/journal.ppat.1004265)
Supplement: Table S8 — Model predicted rates of Fatty acid (FA) and Cholesterol (CL) synthesis in infected cells as ratio of UI cells. (DOCX) [file ppat.1004265.s019.docx]

**Table S8: Model predicted rates of Fatty acid (FA) and Cholesterol (CL) synthesis in infected cells as ratio of UI cells.**

| Rate of Synthesis with respect to UI (Model Prediction) | | | | | | | | | | |
| --- | --- | --- | --- | --- | --- | --- | --- | --- | --- | --- |
|  | H37Ra/UI | | M.smeg/UI | | H37Rv/UI | | JAL2287/UI | | BND433/UI | |
| Hours p-i | CL | FA | CL | FA | CL | FA | CL | FA | CL | FA |
| 6 | 1.5 | 1.2 | 2 | 1.04 | 1 | 0.961 | 7 | 13.6 | 10 | 2.33 |
| 12 | 1.5 | 1.03 | 1.3 | 1.2 | 4.5 | 1.68 | 7 | 8.18 | 10 | 5.33 |
| 24 | 1 | 1.1 | 1.7 | 1.2 | 4 | 2.45 | 4 | 4.58 | 9 | 15 |
| 36 | 1.5 | 1.1 | 1.8 | 1.2 | 4 | 4.1 | 2.2 | 3.94 | 7 | 21 |
| 48 | 1.7 | 1.1 | 1 | 1.1 | 7 | 3.64 | 2 | 4.28 | 8 | 22 |
