# Supplementary material for: Pathogenicity of Mycobacterium tuberculosis Is Expressed by Regulating Metabolic Thresholds of the Host Macrophage
Source: PLoS Pathog. 2014 Jul 24;10(7):e1004265. doi: 10.1371/journal.ppat.1004265 (PMC4110042; doi:10.1371/journal.ppat.1004265)
Supplement: Table S9 — Table showing experimentally determined fatty acid (FA) and cholesterol synthesis (CL) rates as ratios over UI cells. (DOCX) [file ppat.1004265.s020.docx]

**Table S9: Experimentally determined fatty acid (FA) and cholesterol synthesis (CL) rates as ratios over UI cells**

| Rate of Synthesis with respect to UI (Experiment result) | | | | | | | | | | |
| --- | --- | --- | --- | --- | --- | --- | --- | --- | --- | --- |
|  | H37Ra/UI | | M.smeg/UI | | H37Rv/UI | | JAL2287/UI | | BND433/UI | |
| Hours p-i | CL | FA | CL | FA | CL | FA | CL | FA | CL | FA |
| 6 | 1±0.09 | 1.21±0.16 | 2±0.32 | 1.42±0.3 | 0.8±0.07 | 1.09±0.29 | 5.2±0.98 | 14±1.22 | 7±1.12 | 2±0.09 |
| 36 | 0.6±0.08 | 1.17±0.19 | 1.2±0.09 | 1.18±0.48 | 10±1.25 | 3.5±0.49 | 0.8±0.16 | 4±0.38 | 4±0.43 | 18±2.2 |
